# Cep57 is a Mis12-interacting kinetochore protein involved in kinetochore targeting of Mad1–Mad2

**DOI:** 10.1038/ncomms10151

**Published:** 2016-01-08

**Authors:** Haining Zhou, Tianning Wang, Tao Zheng, Junlin Teng, Jianguo Chen

**Affiliations:** 1 Key Laboratory of Cell Proliferation and Differentiation of the Ministry of Education and State Key Laboratory of Membrane Biology, College of Life Sciences, Peking University, Beijing 100871, China; 2 Peking-Tsinghua Center for Life Sciences, Academy for Advanced Interdisciplinary Studies, Peking University, Beijing 100871, China; 3 Center for Quantitative Biology, Peking University, Beijing 100871, China

## Abstract

The spindle assembly checkpoint (SAC) arrests cells in mitosis by sensing unattached kinetochores, until all chromosomes are bi-oriented by spindle microtubules. Kinetochore accumulation of the SAC component Mad1–Mad2 is crucial for SAC activation. However, the mechanism by which Mad1–Mad2 accumulation at kinetochores is regulated is not clear. Here we find that Cep57 is localized to kinetochores in human cells, and binds to Mis12, a KMN (KNL1/Mis12 complex/Ndc80 complex) network component. Cep57 also interacts with Mad1, and depletion of Cep57 results in decreased kinetochore localization of Mad1–Mad2, reduced SAC signalling and increased chromosome segregation errors. We also show that the microtubule-binding activity of Cep57 is involved in the timely removal of Mad1 from kinetochores. Thus, these findings reveal that the KMN network-binding protein Cep57 is a mitotic kinetochore component, and demonstrate the functional connection between the KMN network and the SAC.

## Abstract (web summary)

The spindle assembly checkpoint relies on the accumulation of Mad1-Mad2 at kinetochores, but the mechanism of regulation is not known. Here Zhou *et al*. show that the centrosomal protein Cep57 interacts with the kinetochore proteins Mis12 and Mad1, and regulates the recruitment of Mad1/Mad2 to kinetochores.

## Graphical abstract

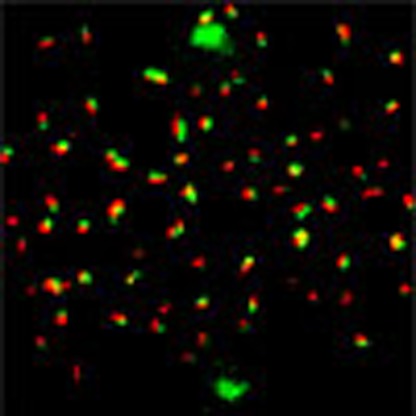

The spindle assembly checkpoint (SAC) arrests cells in mitosis by monitoring kinetochore–microtubule attachment until all chromosomes are bi-oriented on the metaphase plate by spindle microtubules, and ensures accurate chromosome segregation and genomic stability^1^. Unattached kinetochores, as the primary sources of SAC signalling, are considered to be required for the retention of the checkpoint components Mad1 and Mad2 (refs 1, 2). Mad1 binds with itself to form a homodimer, which further binds to two Mad2s, then the Mad1–Mad2 tetramer is concentrated on unattached kinetochores in a Mad1-dependent manner^3^,^4^,^5^. The kinetochore-tethered tetramer acts as a ‘template' for the transformation of cytosolic Mad2 from ‘open' to ‘closed'^6^,^7^. The closed Mad2 binds to Cdc20, and cooperates with BubR1 and Bub3, binding partners of Cdc20, to form the mitotic checkpoint complex that prevents Cdc20-dependent activation of the anaphase-promoting complex/cyclosome (APC/C), which is required for the ubiquitin-mediated degradation of securin and cyclin B1 to initiate anaphase and exit from mitosis^8^,^9^,^10^,^11^,^12^.

Accumulation of Mad1–Mad2 on unattached kinetochores is crucial for SAC signalling^8^. Despite the importance of this process, it is still unclear, precisely, which kinetochore components are responsible for the anchoring^1^,^8^,^13^. Some kinetochore proteins, such as Hec1, Nuf2, CENP-I and the RZZ complex (ROD, ZWILCH and ZW10), have been reported to be involved in regulating Mad1–Mad2 at kinetochores^14^,^15^,^16^,^17^,^18^,^19^,^20^,^21^,^22^. Depletion of Hec1, Nuf2 or CENP-I decreases the kinetochore signal of Mad1 (refs 14, 18, 23), and the RZZ complex component ZW10 is also required for the kinetochore localization of Mad1–Mad2 (refs 15, 17, 19), but none of them has been identified as a direct binding partner of Mad1 or Mad2 (refs 16, 19, 23). Bub1 and Mad1 have been reported to bind to each other in *Caenorhabditis elegans* and yeast^24^,^25^. The KMN (KNL1/Mis12 complex/Ndc80 complex) network is an important scaffold for checkpoint protein tethering^26^,^27^,^28^, and its component KNL1 acts as a recruiter of RZZ and Bub1 (refs 26, 27, 29, 30), whereas the minimal structural elements that recruit Mad1–Mad2 remain to be elucidated.

*Xenopus* Cep57 (*x*Cep57) is associated with Mis12, Zwint-1, Ndc80/Hec1, CLIP170 and microtubules, and is required for the maintenance of spindle microtubule anchorage at kinetochores, while its accumulation at the centrosome is thought to be involved in microtubule nucleation^31^. There are two homologues of *x*Cep57 in mammals, Cep57 (also known as translokin)^32^ and Cep57-related protein (Cep57R), and each shows ∼30% identity with *x*Cep57 (ref. 33). The localization and function of Cep57R remains unknown, while Cep57 is an ubiquitously-expressed protein that functions in spindle assembly, spindle pole integrity and central spindle organization as a microtubule- and centrosome-associated protein^33^,^34^,^35^,^36^. Also, Cep57 is required for centriole duplication, and is highly expressed in prostate cancer cells^37^. Both *x*Cep57 and Cep57 are localized at centrosomes^31^,^33^,^34^,^36^, but whether Cep57 is also localized to kinetochores remains unclear.

Here we show that Cep57 is localized to kinetochores. Interestingly, Cep57 interacts with kinetochore protein Mis12 and the SAC component Mad1. The binding of Cep57 to Mis12 is required for the kinetochore localization of Cep57. Cep57 also binds with Mad1, and is involved in the recruitment of Mad1–Mad2 to kinetochores. Besides, the competition between Mad1 and microtubules for binding to Cep57 functions in removal of Mad1 from kinetochores. Thus, we propose that Cep57 regulates the Mad1–Mad2-mediated monitoring of kinetochore-microtubule attachment.

## Results

### Cep57 is a kinetochore component and binds to Mis12

**Figure 1:**
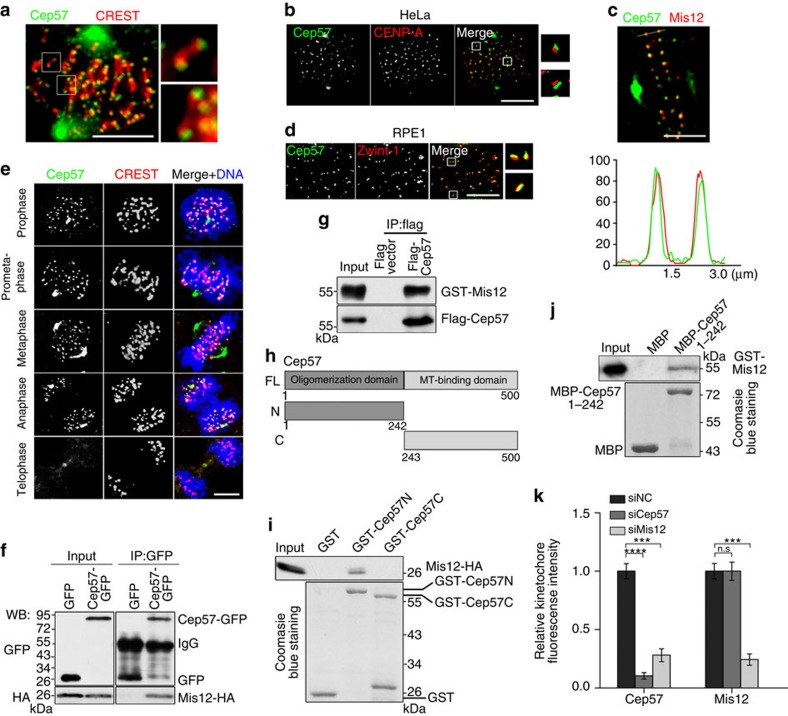
Cep57 is a novel kinetochore component in human cells. (**a**) Three-dimensional structured illumination microscopy (SIM) images of HeLa cells double-immunostained with antibodies against Cep57 (green) and CREST (red). (**b**) Immunofluorescence of Cep57 (green) and CENP-A (red) in HeLa cells. (**c**) Immunofluorescence of Cep57 (green) and Mis12 (red) in HeLa cells at metaphase after treatment with MG132 for 1 h. A linescan through the kinetochore pair indicates the co-localization of Cep57 with Mis12. (**d**) Stimulated emission depletion (STED) images of immunofluorescence of Cep57 (green) and Zwint-1 (red) in RPE1 cells. (**e**) Immunofluorescence of Cep57 (green) and CREST (red) in HeLa cells at different stages during mitosis. DNA was stained with 4,6-diamidino-2-phenylindole (DAPI, blue). (**f**) HEK293T cells were co-transfected with Cep57-GFP and Mis12-HA. The cell lysates were immunoprecipitated (IP) and analysed by western blotting (WB) with the indicated antibodies. (**g**) Binding assays of Mis12 and Cep57. GST-Mis12 (expressed in *E. coli* and purified) was incubated with Flag-Cep57 (expressed in HEK293T cells and purified). The IP samples with anti-Flag antibody were analysed by WB with anti-Flag and anti-Mis12 antibodies. (**h**) Schematic of Cep57 truncated mutants. (**i**) GST pull-down assays of Cep57 and Mis12. Lysates of HEK293T cells overexpressing Mis12-HA were incubated with Glutathione Sepharose 4B beads coated with GST or GST-Cep57N/C. The samples were analysed by WB with anti-HA antibody. GST-tagged proteins were stained with Coomassie blue. (**j**) *In vitro* pull-down assays of Mis12 and Cep57 (1–242 amino acids). GST-Mis12 (expressed in *E. coli* and purified) and MBP-Cep57 (1–242 amino acids) were incubated with Amylose Magnetic beads. The precipitated samples were analysed by WB with anti-GST antibody and Coomassie blue staining. (**k**) Quantification of kinetochore signals of Cep57 and Mis12 in HeLa cells depleted of Cep57, Mis12 or negative control (NC) by siRNAs. The signal from control siRNA-treated cells was normalized to 1.0. More than 200 kinetochores from 20 cells were measured. The experiment was repeated three times. Data are mean±s.e.m. ****P<0.0001; ***P<0.001; NS, not significant (unpaired two-tailed Student's *t*-test). Scale bars, 5 μm in (**a**–**e**).

*x*Cep57 is a kinetochore component^31^. To determine whether Cep57 is also located at kinetochores in human cells, we raised a mouse polyclonal antibody against the Cep57 protein (∼60 kDa; Supplementary Fig. 1a)^34^, which did not cross-react with Cep57R (Supplementary Fig. 1b). We immunostained for Cep57 with this antibody together with the centromere marker CREST^38^ in HeLa cells, and found that Cep57 was localized at kinetochores (Fig. 1a). The kinetochore staining of Cep57 was confirmed with a rabbit polyclonal anti-Cep57 antibody^34^ and a commercially available anti-Cep57 antibody (GeneTex, Irvine, California, USA; Supplementary Fig. 1c). To investigate the kinetochore localization of Cep57 in detail, we further co-immunostained for Cep57 and the inner kinetochore marker CENP-A^39^ or the KMN network component Mis12 (ref. 40). Cep57 appeared adjacent to CENP-A (Fig. 1b), and was co-localized with Mis12 at kinetochores, while Mis12 showed a slightly more interior localization than Cep57 (Fig. 1c). In RPE1 cells, immunofluorescence showed the co-localization of Cep57 with Zwint-1, an outer kinetochore protein^41^ (Fig. 1d). These results suggest that Cep57 is an outer kinetochore protein in human cells. Moreover, Cep57 was localized at kinetochores until the cell entered telophase during the cell cycle (Fig. 1e).

Next, the yeast two-hybrid and co-immunoprecipitation assays showed that like *x*Cep57 (ref. 31), Cep57 interacted with Mis12 (Supplementary Fig. 1d; Fig. 1f). We further used purified Cep57 from HEK293T cells and Mis12 from bacteria to perform immunoprecipitation assays, and also found that they interacted (Fig. 1g). To determine which regions of Cep57 are responsible for the interaction, we constructed glutathione *S*-transferase (GST)-tagged truncated Cep57 mutants (Fig. 1h) and performed pull-down assays. The results showed that the N terminus of Cep57 (1–242 amino acids) interacted with Mis12 (Fig. 1i). Pull-down assays with MBP-Cep57N (1–242 amino acids) and GST-Mis12 (both expressed in bacteria and purified) showed their direct binding *in vitro* (Fig. 1j).

Then, we knocked down Cep57 and Mis12 using RNA interference (RNAi) to examine their functional relationships in HeLa cells. Depletion of either Cep57 or Mis12 did not affect the total protein levels of the other (Supplementary Fig. 1e,f). Cep57 depletion reduced its kinetochore signal by ∼90%, while the Mis12 signal did not change (Fig. 1k; Supplementary Fig. 1e,g). However, depletion of Mis12 not only decreased its own kinetochore localization (by ∼75%) but also decreased the kinetochore localization of Cep57 (by ∼71%; Fig. 1k; Supplementary Fig. 1f,g). Together, these results suggest that Cep57 interacts with Mis12, and Mis12 is indispensable for the kinetochore localization of Cep57.

### Kinetochore targeting of Cep57 via Mis12

**Figure 2:**
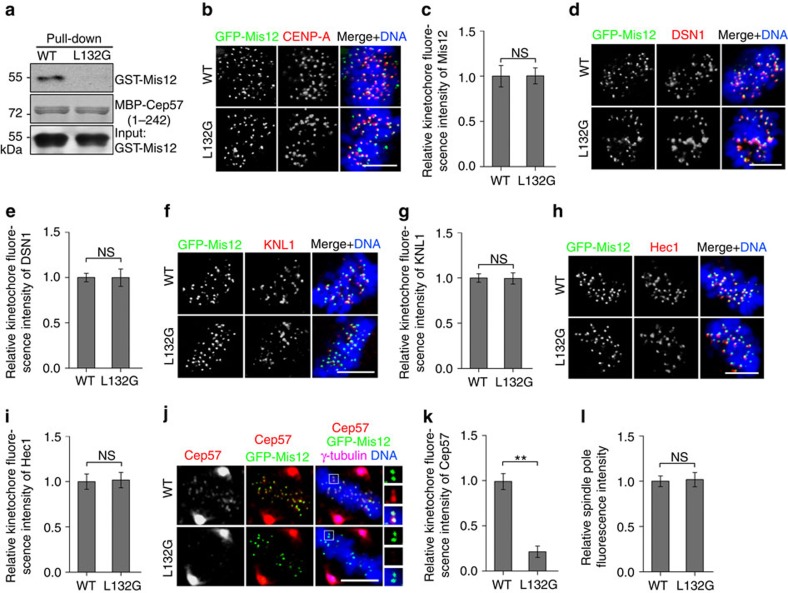
The interaction of Cep57 with Mis12 is important for kinetochore targeting of Cep57. (**a**) *In vitro* pull-down assays. Wild-type (WT) or L132G point-mutated GST-Mis12 expressed in *E. coli* and purified, and was incubated with Amylose Magnetic beads coupled with MBP-Cep57 (1–242 amino acids; expressed in *E. coli* and purified). The precipitated samples were analysed by western blotting with anti-GST antibody (GST-Mis12) and Coomassie blue staining (MBP-Cep57). (**b**,**d**,**f**,**h**) Immunostaining of CENP-A (red, **b**), DSN1 (red, **d**), KNL1 (red, **f**) and Hec1 (red, **h**) in prometaphase HeLa cells expressing RNAi-resistant WT or L132G GFP-Mis12 after treatment with Mis12 siRNA. DNA was stained with 4,6-diamidino-2-phenylindole (DAPI, blue). Scale bars, 5 μm. (**c**,**e**,**g**,**i**) Quantification of the kinetochore signal of GFP-Mis12 (**c**) from **b**, DSN1 (**e**) from **d**, KNL1 (**g**) from **f** and Hec1 (**i**) from **h**. The signal of cells expressing wild-type GFP-Mis12 was normalized to 1.0. More than 100 kinetochores from 10 cells were measured. The experiment was repeated three times. Data are mean±s.e.m.. NS, not significant (unpaired two-tailed Student's *t*-test). (**j**) Immunostaining of Cep57 (red) and γ-tubulin (purple) in metaphase HeLa cells expressing RNAi-resistant WT or L132G GFP-Mis12 after treatment with Mis12 siRNA. DNA was stained with DAPI (blue). Scale bars, 5 μm. (**k**,**l**) Quantification of the kinetochore (**k**) and spindle pole (**l**) signals of Cep57 from (**j**). The signal of cells expressing wild-type GFP-Mis12 was normalized to 1.0. More than 100 kinetochores from 10 cells were measured. The experiment was repeated three times. Data are mean±s.e.m. **P<0.01; NS, not significant (unpaired two-tailed Student's *t*-test).

To further investigate whether the interaction between Mis12 and Cep57 is required for the kinetochore localization of the latter, we sought to disrupt the interaction with mutations in Mis12. *In vitro* pull-down assays using Mis12 mutants and Cep57 (1–242 amino acids; all expressed in *E. coli* and purified) showed that deletion of the amino acids 111–140 region of Mis12 abolished its interaction with Cep57 (Supplementary Fig. 2a,b). In yeast two-hybrid assays, we further narrowed down the region and found that deletion of amino acids 131–140 was sufficient to disrupt the interaction (Supplementary Fig. 2c,d). Then, we set out to determine the critical residues within the region or nearby by mutating some conserved and characteristic amino acids to glycine (Supplementary Fig. 2e). We found that single amino-acid substitution (L132G) in Mis12 was enough to abolish its interaction with Cep57 in yeast two-hybrid assays (Supplementary Fig. 2f); this was confirmed by *in vitro* pull-down assays with recombinant Mis12 point mutant (L132G) and Cep57 (1–242 amino acids) expressed in *E. coli* and purified (Fig. 2a). The L132G mutation of Mis12 did not markedly affect its kinetochore localization and that of some other KMN network components (DSN1, KNL1 and Hec1; Fig. 2b–i), which had been considered to require Mis12 for their kinetochore targeting^40^. However, the L132G mutant specifically led to decrease in the localization of Cep57 at kinetochores (by ∼79%), but not at spindle poles (Fig. 2j–l), suggesting that interaction with Mis12 is required for Cep57 to efficiently anchor kinetochores.

### Cep57 is involved in activation of the mitotic checkpoint

**Figure 3:**
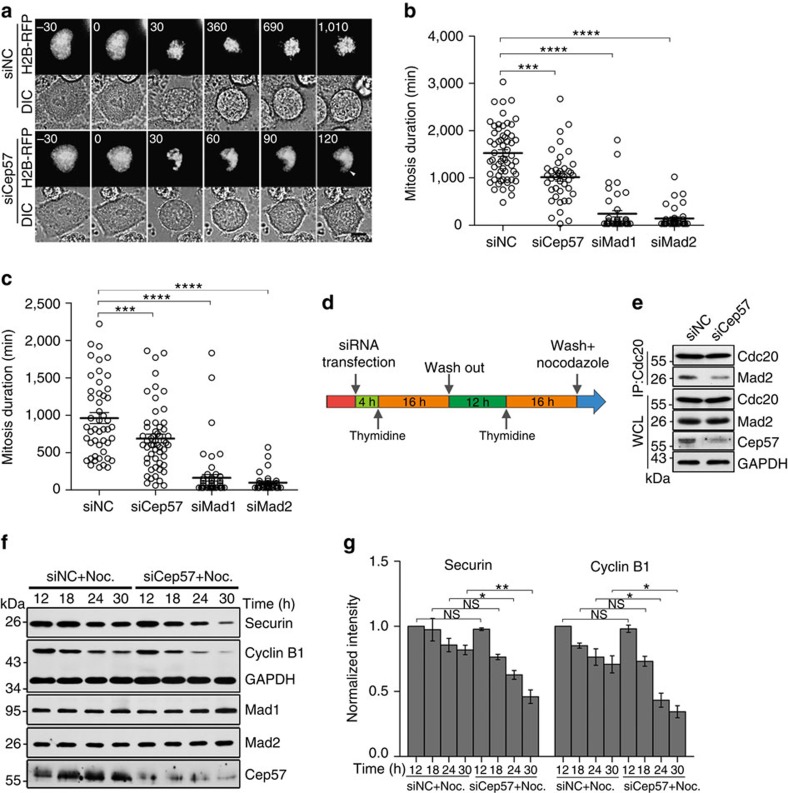
Cep57 contributes to activation of the spindle assembly checkpoint. (**a**,**b**) HeLa cells with 100 nM nocodazole treatment after co-transfection with H2B-RFP and negative control (NC)-, Cep57-, Mad1- or Mad2-siRNA for 48 h. (**a**) Time-lapse images of HeLa cells. The numbers indicate the time (minutes) after entry into mitosis. Arrowhead, multiple nuclei. Scale bar, 5 μm. (**b**) Quantification of mitotic duration of HeLa cells from **a**. Scatter plots show data from three independent experiments. (**c**) Quantification of mitotic duration of live HeLa cells with 80 nM taxol treatment after co-transfection with H2B-RFP and negative control (NC)-, Cep57-, Mad1- or Mad2-siRNA for 48 h. Scatter plots show data from three independent experiments. (**d**) Diagram of experimental design. The siRNA-transfected HeLa cells were synchronized by double-thymidine block, and released into nocodazole. (**e**–**g**) HeLa cells were treated as in **d**. (**e**) After 12 h nocodazole treatment, mitotic cell lysates were used to perform immunoprecipitation (IP) and western blotting assays with the indicated antibodies. WCL, whole-cell lysate. (**f**) Western blots of the indicated proteins in HeLa cells after the indicated nocodazole (Noc.) treatment time. (**g**) Quantification of the protein levels of securin and cyclin B1 from (**f**). The experiment was repeated three times. For **b**,**c** and **g**, data are mean±s.e.m. ****P<0.0001; ***P<0.001; **P<0.01; *P<0.05; NS, not significant (unpaired two-tailed Student's *t*-test). DIC, differential interference contrast.

The KMN network is an important scaffold for the kinetochore accumulation of SAC components^1^,^26^,^27^. To determine whether Cep57, a KMN-associated protein, functions in the SAC, we monitored the mitotic progression of cells transfected with Cep57-siRNA followed by nocodazole treatment, which induced long-term activation of the SAC by unattached kinetochores in mitotic cells. Control HeLa cells were arrested in mitosis for a median time of 1,524 min (Fig. 3a,b; Supplementary Movie 1), while in Cep57-depleted cells, the time was reduced to 1,016 min (Fig. 3a,b; Supplementary Movie 2); a reduction also occurred in Mad1- or Mad2-depleted cells (Fig. 3b; Supplementary Fig. 3a,b). Similar results were obtained when cells were transfected with Cep57-siRNA and treated with taxol, a drug that inhibited spindle dynamics and activated the SAC (Fig. 3c). Furthermore, the mitotic index was decreased in Cep57-depleted cells after treatment with nocodazole (10–100 nM; Supplementary Fig. 3c). Under treatment with 100 nM nocodazole, the mitotic index of Cep57-depleted cells was reduced to ∼49% from ∼77% in control cells, and siRNA-resistant Cep57 rescued this index to ∼65% (Supplementary Fig. 3d,e). Consistently, we found an increased percentage of cells with multiple small nuclei after Cep57 depletion, and this was also rescued by siRNA-resistant Cep57 (Supplementary Fig. 3f,g). Collectively, these data suggest that depletion of Cep57 attenuates the SAC activation induced by drugs that affect microtubules.

Unattached kinetochores trigger a conformational change in cytoplasmic Mad2, and induce its binding to Cdc20 (ref. 8). This binding prevents the activation of APC/C and the degradation of its substrates^10^,^12^. Therefore, we investigated whether the weakened mitotic arrest in Cep57-depleted cells is mediated by the inhibition of Mad2-dependent Cdc20-APC/C. HeLa cells were synchronized by double-thymidine block and released into nocodazole after siRNA transfection (Fig. 3d). After released into nocodazole for 12 h, the mitotic cells were collected for immunoprecipitation with anti-Cdc20 antibody. The results showed that Cep57 depletion decreased the binding of Cdc20 to Mad2 (Fig. 3e). Then, after double-thymidine block, we collected cells after different durations of treatment with nocodazole to probe the APC/C substrates securin and cyclin B1. Compared with control cells, Cep57-depleted cells showed a more rapid decrease in the protein levels of both securin and cyclin B1 (Fig. 3f,g), indicating that Cep57 depletion relieves the nocodazole-induced and Mad2-dependent inhibition of APC/C.

### Role of Cep57 in mitotic progression/chromosome segregation

**Figure 4:**
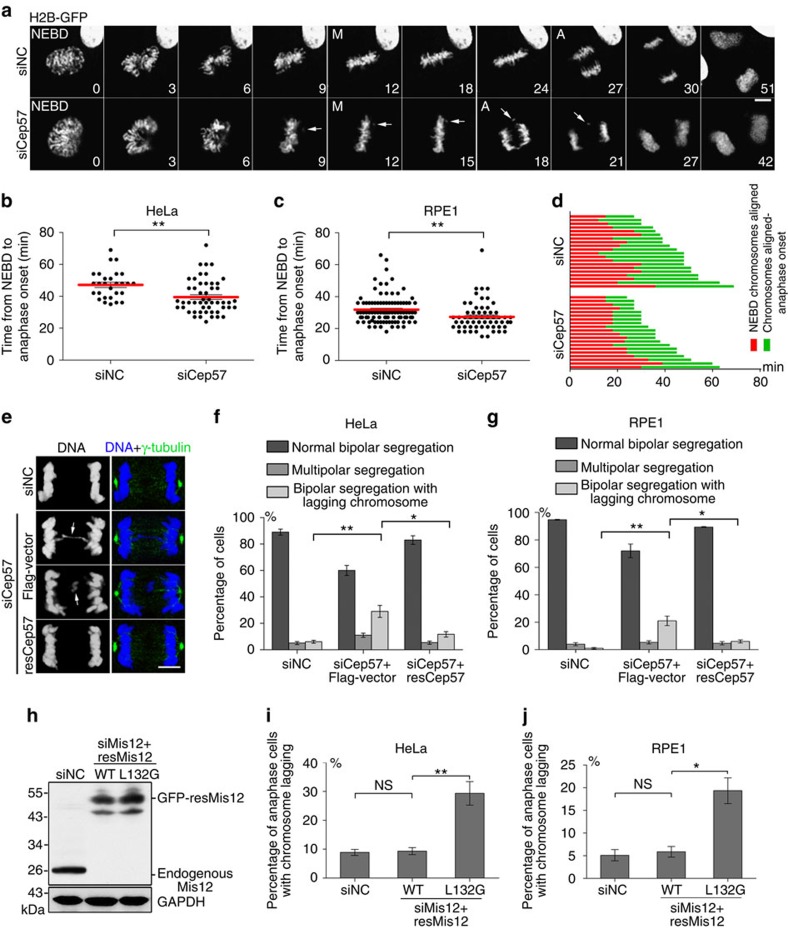
Cep57 depletion accelerates anaphase onset and leads to chromosome lagging. (**a**) Time-lapse images of RPE1 cells co-transfected with H2B-GFP and negative control (NC)- or Cep57-siRNA for 60 h. NEBD, nuclear envelope breakdown; M, metaphase; A, anaphase. The numbers indicate the time (minutes) after NEBD. Arrows, misaligned chromosomes and lagging chromosomes. Scale bar, 5 μm. (**b**,**c**) Quantification of time from NEBD to anaphase onset of HeLa (**b**) and RPE1 cells (**c**) co-transfected with H2B-GFP and NC- or Cep57-siRNA for 60 h. Scatter plots show data from three independent experiments (red lines indicate the average). Data are mean±s.e.m. **P<0.01 (unpaired two-tailed Student's *t*-test). (**d**) Mitotic progression from NEBD to anaphase onset in HeLa cells transfected with H2B-RFP and NC- or Cep57-siRNA for 60 h. The cells were selected at random. (**e**) Immunostaining of γ-tubulin (green) in HeLa cells with NC-siRNA, Cep57-siRNA or Cep57-siRNA and siRNA-resistant Cep57 (resCep57) for 60 h. DNA was stained with 4,6-diamidino-2-phenylindole (DAPI, blue). Scale bar, 5 μm. Arrows, lagging chromosomes. (**f**,**g**) Percentages of anaphase HeLa (**f**) and RPE1 cells (**g**) after transfection with NC-siRNA, Cep57-siRNA or Cep57-siRNA and siRNA-resistant Cep57 (resCep57) for 60 h. The experiments were repeated three times. Data are mean±s.e.m. **P<0.01; *P<0.05 (unpaired two-tailed Student's *t*-test); *n*>200 cells per experiment. (**h**) Western blots show endogenous Mis12 depletion in HeLa cells after twice transfection of siRNA for 48 h, and rescue by expressing siRNA-resistant GFP-tagged wild-type (WT) or mutant L132G Mis12 (resMis12). (**i**,**j**) Percentages of anaphase HeLa (**i**) and RPE1 cells (**j**) after twice transfection of Mis12 siRNA and rescue by expressing siRNA-resistant GFP-tagged wild-type (WT) or mutant L132G Mis12 (resMis12). The experiments were repeated three times. Data are mean±s.e.m. **P<0.01; *P<0.05; NS, not significant (unpaired two-tailed Student's *t*-test); *n*>200 cells per experiment.

Activation of the SAC delays anaphase initiation and ensures the equal distribution of chromosomes into two daughter cells^1^,^2^. To further determine whether Cep57, functioning during nocodazole-induced SAC activation, contributes to mitotic progression and chromosome segregation, we monitored Cep57-depleted HeLa and RPE1 cells by live-cell imaging microscopy, and found that with Cep57 depletion the average time from nuclear envelope breakdown to anaphase onset was shortened by 7 min in HeLa cells, and by 4 min in RPE1 cells relative to controls (Fig. 4a–c; Supplementary Fig. 4a,b; Supplementary Movie 3–6). We further found that the metaphase time (from the point of chromosome alignment to anaphase onset) was decreased in Cep57-depleted cells, but the chromosome alignment time (from nuclear envelope breakdown to the point of chromosome alignment) was not (Fig. 4a,d; Supplementary Fig. 4a–c; Supplementary Movie 3–6). These data suggest that Cep57 depletion induces the premature onset of anaphase.

Consistent with the appearance of a premature metaphase–anaphase transition, chromosome lagging occurred more frequently in Cep57-depleted cells than in controls; it was elevated by 23% in HeLa cells, and by 17% in RPE1 cells (Fig. 4a; Supplementary Fig. 4a,d; Supplementary Movie 3–6). Considering that Cep57 is also localized to spindle poles, and its depletion results in an increased percentage of cells with multipolar spindles^34^, which may also induce chromosome lagging. To define whether kinetochore-localized Cep57 contributes to avoiding chromosome segregation errors, we first labelled γ-tubulin in HeLa cells transfected with Cep57-siRNA and calculated the percentage of bipolar segregated cells with chromosome lagging, and found that it was significantly raised by ∼24%, and the siRNA-resistant Cep57 restored it by ∼15% (Fig. 4e,f). Similar results were obtained in RPE1 cells (Fig. 4g; Supplementary Fig. 4b). Furthermore, the point mutant of Mis12 (L132G) that specifically reduced the kinetochore-localized Cep57 (Fig. 2j–l) increased the frequency of chromosome lagging in both HeLa (by ∼19%) and RPE1 cells (by ∼13%) but not that of multipolar spindles (Fig. 4h–j; Supplementary Fig. 4e–g).

Taken together, our results suggest that Cep57 is required for mitotic timing control and correct chromosome segregation.

### Cep57 contributes to the recruitment of Mad1 to kinetochores

**Figure 5:**
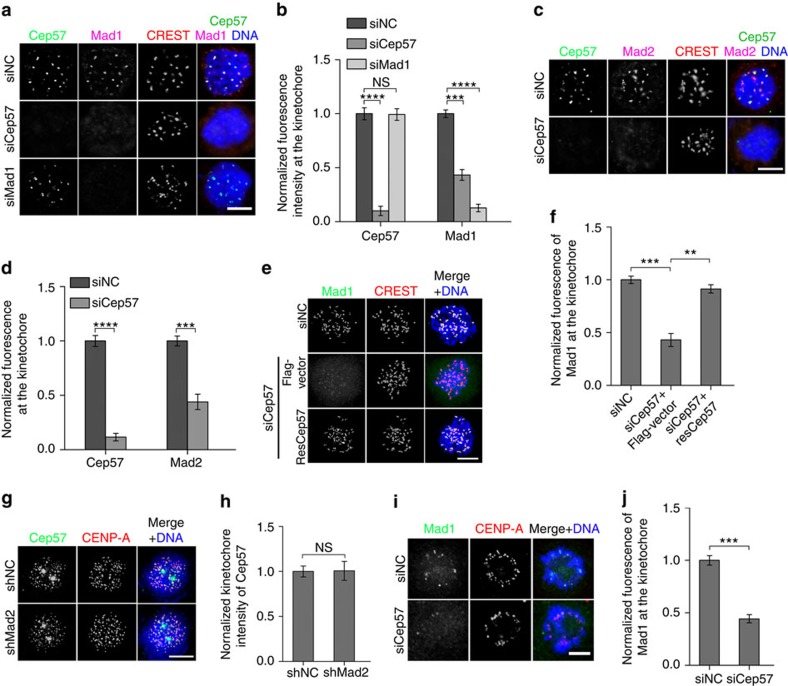
Cep57 depletion reduces kinetochore anchoring of Mad1–Mad2. (**a**–**d**) Depletion of the indicated proteins in HeLa cells by siRNAs for 60 h followed by treatment with nocodazole and MG132 for 1 h. NC, negative control. Immunostaining of Cep57 (green), Mad1 (purple) and CREST (red, **a**); and Cep57 (green), Mad2 (purple) and CREST (red, **c**). DNA was stained with 4,6-diamidino-2-phenylindole (DAPI, blue). Scale bars, 5 μm. Cep57 and Mad1 (**b**) signals; and Cep57 and Mad2 (**d**) signals in cells from **a** and **c**, respectively, were normalized against CREST. The experiment was repeated three times. (**e**) Immunostaining of Mad1 (green) and CREST (red) in HeLa cells transfected with the indicated siRNAs and vectors for 60 h followed by nocodazole and MG132 treatment for 1 h. ResCep57, siRNA-resistant Cep57. DNA was stained with DAPI (blue). Scale bar, 5 μm. (**f**) Mad1 signal in cells from **e** was normalized against CREST. The experiment was repeated three times. (**g**) Immunostaining of Cep57 (green) and CENP-A (red) in HeLa cells transfected with the indicated shRNAs. DNA was stained with DAPI (blue). Scale bar, 5 μm. (**h**) Quantification and normalization of the kinetochore signal of Cep57 from **g**. The experiment was repeated three times. (**i**) Immunostaining of Mad1 (green) and CENP-A (red) in HeLa cells transfected with NC- or Cep57-siRNA for 60 h followed by treatment with 80 nM taxol and MG132 for 4 h. DNA was stained with DAPI (blue). Scale bar, 5 μm. (**j**) Quantification and normalization of the kinetochore signal of Mad1 from **i**. The experiment was repeated three times. For **b**,**d**,**f**,**h** and **j**, >200 kinetochores from 20 cells were measured per experiment. Data are mean±s.e.m. ****P<0.0001; ***P<0.001; **P<0.01; NS, not significant (unpaired two-tailed Student's *t*-test).

SAC signalling is considered to be initiated by the accumulation of the Mad1–Mad2 complex at kinetochores^4^,^8^, so we tested whether Cep57 is necessary for the kinetochore recruitment of this complex. Cep57 depletion not only reduced its own kinetochore signal by ∼90%, but also the signal of Mad1 (by ∼53%) and Mad2 (by ∼51%) in HeLa cells with nocodazole treatment (Fig. 5a–d), though the total protein levels of Mad1 and Mad2 did not change (Supplementary Fig. 5a). SiRNA-resistant Cep57 restored the kinetochore signal of Mad1 to ∼88% (Fig. 5e,f). However, neither Mad1 nor Mad2 depletion affected the either protein level or kinetochore localization of Cep57 (Fig. 5a,b,g,h; Supplementary Fig. 5b,c). In taxol-treated cells, the kinetochore-targeting efficiency of Mad1 was also reduced by Cep57 depletion (by ∼51%; Fig. 5i,j). Thus, Cep57 is involved in the kinetochore recruitment of Mad1–Mad2.

To determine whether the responsibility of Cep57 for the kinetochore anchoring of Mad1–Mad2 is specific, we tested some other kinetochore components, KNL1 (refs 26, 27), Zwint-1 (refs 42, 43), ZW10 (refs 19, 21), Bub1 (refs 1, 2, 16) and BubR1 (refs 1, 2, 8). Their kinetochore localization was not significantly affected by Cep57 depletion (Supplementary Fig. 5d–m), suggesting that Cep57 specifically functions in Mad1–Mad2 recruitment.

### Cep57 interacts with Mad1

**Figure 6:**
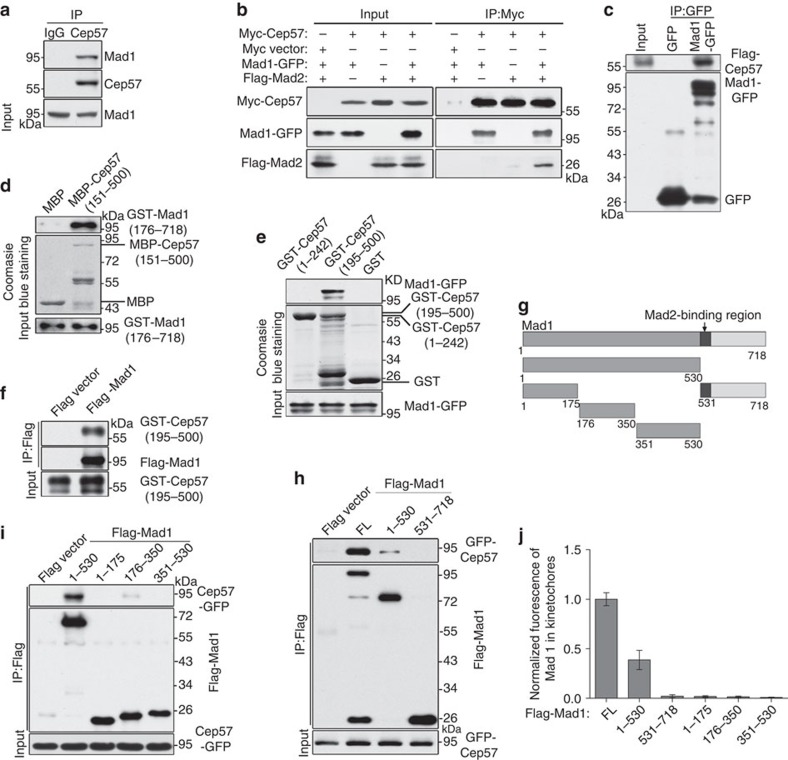
Cep57 interacts with Mad1. (**a**) Mitotic HeLa cells arrested by nocodazole were used for immunoprecipitation (IP) with anti-Cep57 antibody and western blotting with anti-Cep57 and anti-Mad1 antibodies. IgG served as the negative control. (**b**) HEK293T cells were co-transfected with the indicated plasmids, and were used to for IP and western blotting. (**c**) Binding assays of Mad1 and Cep57. Flag-Cep57 and Mad1-GFP (expressed in HEK293T cells and purified) were incubated for IP with anti-GFP antibody. IP samples were analysed by western blotting with anti-Flag and anti-GFP antibodies. (**d**) *In vitro* pull-down assays of Mad1 and Cep57. GST-Mad1 (176–718 amino acids) and MBP-Cep57 (151–500 amino acids) (expressed in *E. coli* and purified) were incubated with Amylose Magnetic beads. The precipitated samples were analysed by western blotting with anti-GST antibody and Coomassie blue staining. (**e**) GST pull-down assays of Cep57 and Mad1. Lysates of HEK293T cells overexpressing Mad1-GFP were incubated with Glutathione Sepharose 4B beads coated with GST, GST-Cep57 (1–242 amino acids) or GST-Cep57 (195–500 amino acids). The samples were analysed by western blotting with anti-GFP antibody. GST and GST-tagged proteins were stained with Coomassie blue. (**f**) Binding assays of Mad1 and Cep57 (195–500 amino acids). GST-Cep57 (195–500 amino acids; expressed in *E. coli*) and Flag-Mad1 (expressed in HEK293T cells) were purified and incubated with anti-Flag antibody. The IP samples with anti-Flag antibody were analysed by western blotting with anti-GST and anti-Flag antibodies. (**g**) Schematic of truncated mutants of Mad1. (**h**,**i**) IP using lysates of HEK293T cells co-overexpressing GFP-Cep57 and Flag-Mad1 (FL, 1–530 and 531–718 amino acids) (**h**) or Flag-Mad1 (1–530, 1–175, 176–350 and 351–530 amino acids) (**i**) with anti-Flag antibody. The IP samples were analysed by western blotting with anti-GFP and anti-Flag antibodies. FL, full length. (**j**) Quantification and normalization of the kinetochore signal of Flag-Mad1 in HeLa cells that were transfected with FL and truncated mutants of Flag-Mad1 and treated with nocodazole for 1 h. Greater than 50 kinetochores from 5 cells were measured. The experiment was repeated three times. Data are mean±s.e.m.

To investigate the mechanism by which Cep57 is responsible for the kinetochore accumulation of Mad1–Mad2, we determined whether Cep57 was associated with Mad1–Mad2 using yeast two-hybrid assays, and the results showed that Mad1 bound to Cep57 (Supplementary Fig. 6a,b). Immunoprecipitation assays with both endogenous and exogenous proteins also showed the interaction between Cep57 and Mad1 (Fig. 6a; Supplementary Fig. 6c). The interaction of Cep57 with Mad2 was barely detectable unless Mad1 was present (Fig. 6b), which suggested that Cep57 is associated with the Mad1–Mad2 complex via Mad1. We further used purified recombinant Cep57 and Mad1 from HEK293T cells (Fig. 6c) and *E. coli* (Fig. 6d) to perform binding assays and the results showed that Cep57 directly bound to Mad1 *in vitro*. To determine which regions of Cep57 are responsible for the binding, we performed pull-down assays using truncated mutants of Cep57, and found that its C terminus (195–500 amino acids) interacted with Mad1 (Fig. 6e). Purified GST-Cep57 (195–500 amino acids) and Flag-Mad1 co-immunoprecipitated, further confirming the interaction of the C-terminal region of Cep57 with Mad1 (Fig. 6f). We also constructed truncated mutants of Mad1 to map the regions responsible for the binding to Cep57 (Fig. 6g). Immunoprecipitation assays showed that the N terminus of Mad1 (1–530 amino acids) was crucial for this interaction (Fig. 6h). Furthermore, short truncated mutants of the N terminus of Mad1 (1–175 and 351–530 amino acids) barely precipitated with Cep57, and the internal region mutant (176–350 amino acids) also showed a very weak binding affinity (Fig. 6i). In addition, among these mutants of Mad1, only the N-terminal mutant (1–530 amino acids) showed weak kinetochore localization, but its three short truncated mutants (1–175, 176–350 and 351–530 amino acids) or the C terminus (531–718 amino acids) did not show the localization (Fig. 6j; Supplementary Fig. 6d), suggesting that the structural integrity of Mad1 is important for its kinetochore targeting.

### Role of Cep57 microtubule-binding activity in SAC silencing

**Figure 7:**
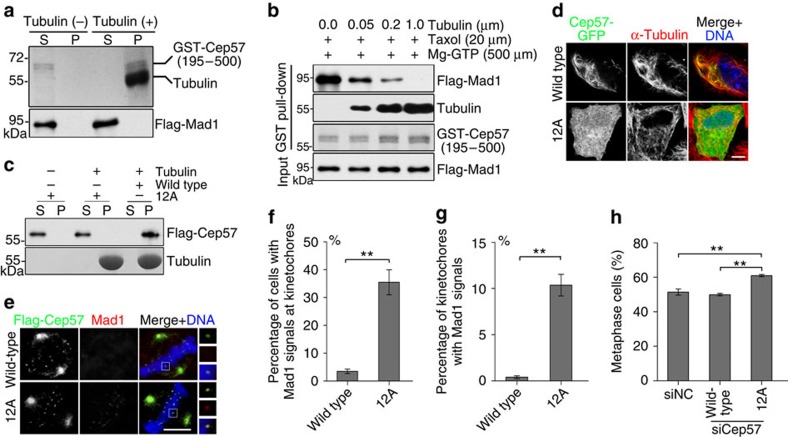
Microtubule-binding activity of Cep57 contributes to checkpoint silencing. (**a**) Microtubule-binding assays *in vitro*. GST-Cep57 (195–500 amino acids; 0.1 μM) expressed in *E. coli* and Flag-Mad1 (0.05 μM) expressed in HEK293T cells were purified and incubated with or without taxol-stabilized microtubules (1.0 μM) in BRB80 buffer. After centrifugation, supernatant (S) and pellet (P) were separated and used for Coomassie blue staining (top), and western blotting with anti-Flag antibody (bottom). (**b**) GST-Cep57 (195–500 amino acids; 0.1 μM)-coupled Glutathione Sepharose 4B beads were incubated with taxol-stabilized microtubules and purified Flag-Mad1 (0.05 μM) in BRB80 buffer at room temperature. The bead-bound proteins were analysed by western blotting with anti-Flag and anti-tubulin antibodies. GST-Cep57 (195–500 amino acids) was detected by Coomassie blue staining. (**c**) Microtubule-binding assays *in vitro*. Flag-Cep57 (0.05 μM) and Flag-Cep57-12A (0.05 μM) expressed in HEK293T cells and purified, and were incubated with or without taxol-stabilized microtubules (1.0 μM) in BRB80 buffer. Samples were separated by centrifugation, and analysed by western blotting with anti-Flag antibody (top) and Coomassie blue staining (bottom). 12A: K432A, K434A, K435A, K438A, K441A, K442A, K467A, R469A, K473A, R474A, R475A and K476A. (**d**) Immunostaining of α-tubulin (red) in HeLa cells expressing Cep57-GFP or Cep57-12A-GFP. DNA was stained with 4,6-diamidino-2-phenylindole (DAPI, blue). Scale bars, 5 μm. (**e**) Immunostaining of Flag-Cep57 (green) and Mad1 (red) in metaphase HeLa cells expressing RNAi-resistant wild-type Flag-Cep57 or Flag-Cep57-12A after transfection with Cep57-siRNA. DNA was stained with DAPI (blue). Scale bars, 5 μm. (**f**) Quantification of the percentage of metaphase cells with Mad1 signals at kinetochores from (**e**). Fifty cells were measured. (**g**) Quantification of the percentage of kinetochores with Mad1 signals in metaphase cells from (**e**). Greater than 100 kinetochores from 10 cells were measured. (**h**) Quantification of the percentage of metaphase cells in negative control (NC) or Cep57-depleted prometaphase and metaphase HeLa cells that expressed RNAi-resistant wild-type Flag-Cep57 or Flag-Cep57-12A. Mitotic stages were quantified by the morphology of DNA and spindles. Greater than 100 cells were measured. For **f**,**g** and **h**, the experiment was repeated three times. Data are mean±s.e.m. **P<0.01 (unpaired two-tailed Student's *t*-test).

Given that Cep57 is a microtubule-binding protein, the binding is mediated by its C terminus^33^, and the same region binds to Mad1 (Fig. 6e,f), we sought to investigate whether the interaction of Cep57 with Mad1 is affected by the binding of Cep57 to microtubules. First, we performed microtubule co-sedimentation assays with purified Cep57, Mad1 and tubulin *in vitro*. The C terminus of Cep57 showed distinct co-sedimentation with microtubules (Supplementary Fig. 7a), whereas Mad1 was not detected in the co-sediment (Fig. 7a). Then, we coupled the C-terminal Cep57 (expressed in bacteria and purified) to beads and performed pull-down assays *in vitro*. The interaction of Cep57 with Mad1 was reduced with increased binding of Cep57 to microtubules (Fig. 7b), indicating that the C-terminal Cep57 strongly binds to microtubules, and this binding inhibits the interaction of Cep57 with Mad1. These results suggest that microtubules competitively replace Mad1 binding to Cep57. In addition, the interaction of Cep57 with Mis12 did not alter the binding activity between Mad1 and Cep57 (Supplementary Fig. 7b), and the binding of Cep57 to microtubules did not affect the interaction of Cep57 with Mis12 (Supplementary Fig. 7c).

To further determine whether the microtubule-binding activity of Cep57 is required for checkpoint silencing in human cells, we generated a mutated Cep57, in which 12 positively charged residues in the C terminus were replaced with alanine (named Cep57-12A) to abolish its microtubule-binding activity and retain its interaction with Mad1 (Fig. 7c, Supplementary Fig. 7d). This mutant was not co-localized with microtubules and diffused throughout the cytoplasm of interphase HeLa cells after overexpression (Fig. 7d), while its kinetochore localization was not significantly reduced compared with that of the wild type in metaphase cells (Supplementary Fig. 7e,f). In Cep57-12A-expressing cells after endogenous Cep57 depletion, we still observed the Mad1-positive immunostaining signal at the metaphase kinetochores, but the signal almost disappeared at this stage in the wild-type Cep57-expressing cells (Fig. 7e–g). Furthermore, with Cep57-12A expression, the percentage of metaphase cells in prometaphase and metaphase cells was increased (by ∼11% relative to wild-type Cep57 expression; Fig. 7h). Together, these data suggest that the loss of microtubule-binding activity of Cep57 delays the removal of Mad1 from kinetochores and results in extended metaphase arrest.

## Discussion

**Figure 8:**
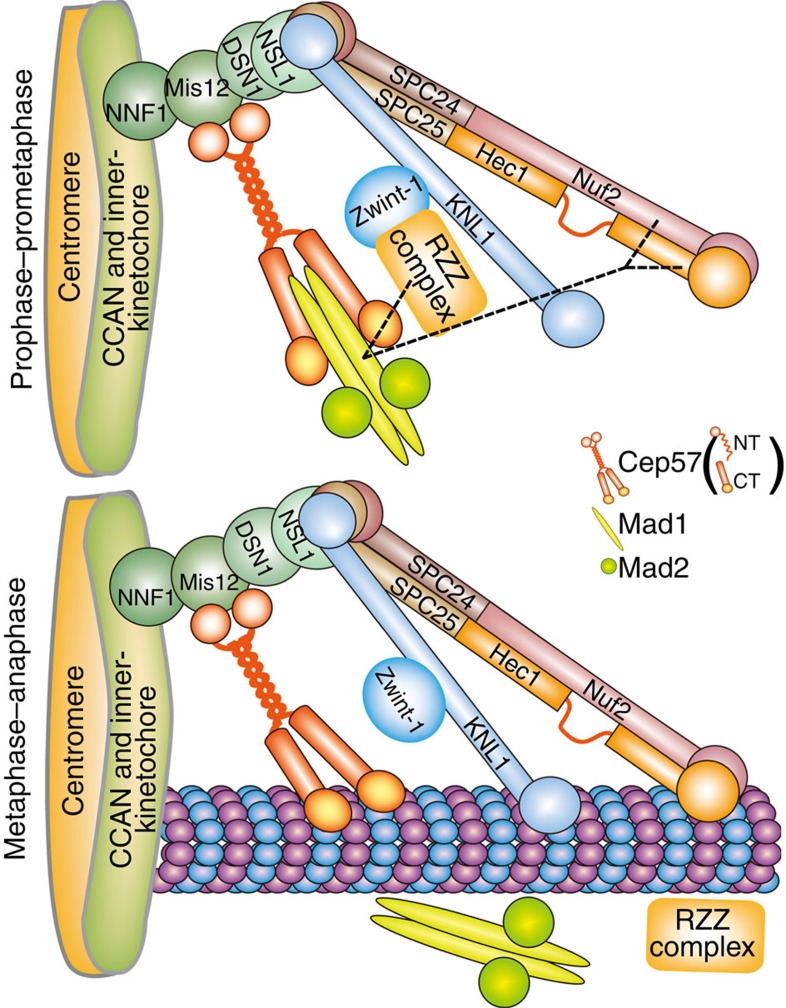
Schematic model. Cep57 is localized to kinetochores and interacts with Mis12 and Mad1. During the early stage of mitosis, the kinetochores are not attached by microtubules and induce SAC activation. The RZZ and Ndc80/Hec1 complexes are responsible for the accumulation of Mad1–Mad2 to kinetochores by direct or indirect means in human cells^14^,^15^,^19^,^23^. Cep57 also acts as one of the anchoring factors of the Mad1–Mad2 complex by the interaction with Mad1 at the kinetochores (top). When the kinetochores are captured by microtubules, Cep57 binds to microtubules along with other outer kinetochore proteins including Hec1, Nuf2 and KNL1 (refs 1, 30). Thus, Mad1–Mad2 is disassociated from the kinetochores, which results in SAC silencing (bottom). NT, N terminus. CT, C terminus.

In this paper, we show that Cep57 is localized to kinetochores, and its N terminus binds to Mis12. Cep57 also interacts with Mad1 via its C terminus and participates in the accumulation of Mad1–Mad2 at kinetochores, while the microtubule-binding activity of Cep57 may contribute to the timely removal of Mad1 from kinetochores (Fig. 8).

We previously reported that Cep57 is a component of the spindle pole and midzone, and functions in spindle pole architecture and central spindle microtubule organization^34^,^35^. Previous findings have revealed that *x*Cep57 is a kinetochore component^31^. Mass spectrometry has shown that chicken Cep57 occurs in isolated mitotic chromosomes^44^. We show here that human Cep57 is localized at kinetochores, suggesting the conservation of kinetochore localization in the Cep57 family.

At kinetochores, the KMN network is considered to contain core microtubule-binding sites^1^. Here we provide evidence that Cep57, as a KMN network-binding protein, is involved in the kinetochore recruitment of Mad1–Mad2 in human cells. Our results showed that significant depletion of Cep57 (∼90%) still left Mad1–Mad2 signals (∼50%) at kinetochores (Fig. 5a–h), indicating that Cep57 is not the unique kinetochore recruiter of Mad1–Mad2. In fact, multiple proteins are involved in the kinetochore accumulation of Mad1–Mad2, and the underlying molecular mechanisms are thought to be complicated and to vary in different species^14^,^23^,^24^,^25^,^45^. In *Caenorhabditis elegans* and yeast, Mad1 has been shown to interact with Bub1, which contributes to the kinetochore accumulation of Mad1 in *Caenorhabditis elegans* cells^24^,^25^. In human cells, Hec1 is required for kinetochore localization of Mad1–Mad2 (refs 23, 46). It has been identified as a Mad1-interacting candidate by a yeast two-hybrid screen, but recombinant Hec1 and Mad1 proteins barely bind to each other *in vitro*^23^. Nuf2, a binding partner of Hec1 and a subunit of the Ndc80/Hec1 complex, has also been reported to function in the kinetochore accumulation of Mad1–Mad2 (refs 14, 46). Though there is no evidence of an interaction between Nuf2 and Mad1–Mad2, depletion of Nuf2 reduces Mad1–Mad2 at kinetochores^14^. Moreover, depletion of the RZZ complex protein ZW10 has also been found to weaken Mad1–Mad2 signals at kinetochores^19^. Similar to Nuf2, ZW10 is not a direct binding partner of Mad1–Mad2, but it may affect the kinetochore localization of Mad1, as Mad1-binding site(s) created by related kinetochore component(s)^19^. Cep57 is a Mis12-binding protein that is closely localized to RZZ and Ndc80/Hec1 complexes. *x*Cep57 is found to show interaction with Ndc80/Hec1 and Zwint-1 (a binding factor and a kinetochore recruiter of ZW10) in *Xenopus*^43^,^47^. So it is possible that Hec1, Nuf2, Bub1, ZW10, Cep57 and other related proteins cooperate to recruit Mad1–Mad2 at the outer kinetochores. This speculation is also supported by previous^48^ and our results that the structural integrity of Mad1 is essential for its fully efficient kinetochore targeting. The N terminus of Mad1 (1–530 amino acids) is essential for kinetochore localization, but the localization is weak, and its shorter truncated mutants are barely detectable at kinetochores. The C terminus of Mad1 (531–718 amino acids) is not localized to kinetochores, but is critical for the efficient accumulation of Mad1 at kinetochore, in line with the report that deletion of the C-terminal domain diminishes Mad1 kinetochore targeting^48^. Therefore, it is likely that multiple regions of Mad1 are required for its kinetochore accumulation via multiple recruiters. Whether Hec1, Nuf2, ZW10, Bub1, Cep57 and even other proteins at the outer kinetochores cooperate to participate in the kinetochore targeting of Mad1–Mad2 in human cells is an interesting issue.

Our results show that Cep57 is associated with Mad2 via Mad1. While in yeast two-hybrid assays, the yeast expressing both bait-Cep57 and prey-Mad2 proliferated slowly in selective medium (Supplementary Fig. 6a), suggesting a possible weak direct interaction between Cep57 and Mad2, though we could not exclude the possibility that yeast Mad1 mediated the interaction. The current model of the SAC activation shows that closed Mad2 is recruited to unattached kinetochores by Mad1, and acts as a catalytic template to trigger the transition from cytoplasmic open Mad2 to closed Mad2 (refs 1, 2, 6, 8, 10). However, it is still unclear how kinetochore-localized Mad2 induces the transformation of cytoplasmic Mad2, and whether other factors are required for Mad2 to function as a template^49^,^50^. The possible connection between Cep57 and Mad2 needs further investigation.

Checkpoint silencing is mainly induced by the removal of essential checkpoint components (such as Mad1, Mad2 and BubR1) from kinetochores, and these components may be driven forward to the spindle pole along kinetochore-connected microtubules by the dynein–dynactin complex and other related proteins^51^,^52^,^53^,^54^,^55^,^56^. When the spindle microtubules are searching for kinetochores and the kinetochore–microtubule attachment has not been fully established^1^, the kinetochore-localized Cep57 may facilitate its interaction with Mad1, but not microtubules, to contribute to the accumulation of Mad1–Mad2, though Cep57 shows strong microtubule-binding activity (Fig. 7a,b)^33^. When kinetochores have been attached by microtubules, Mad1–Mad2 is dissociated from kinetochores by unknown mechanisms^1^,^2^,^57^, although KNL1– and Ndc80/Hec1 complex–microtubule interactions and protein modifications such as dephosphorylation are involved in the SAC silencing^28^,^58^,^59^,^60^. Considering our results that Cep57 shows stronger binding activity to microtubules than to Mad1, it is possible that with the establishment of kinetochore–microtubule attachment, the kinetochore-localized Cep57 binds to microtubules, which facilitates the dissociation of Mad1–Mad2 from kinetochores and contributes to the SAC silencing.

Many kinetochore proteins (such as Hec1, Nuf2 and KNL1) show microtubule-binding activity, and they may have diverse and redundant mechanisms to ensure proper microtubule–kinetochore attachment. Hec1 and Nuf2 are thought to be the most important for load-bearing attachments and microtubule plus-end dynamics, since their N termini have a calponin-homology microtubule-binding domain that is similar to the plus-end tracking protein EB1 (end-binding 1)^1^,^30^,^61^. However, KNL1 microtubule-binding activity, achieved by a short motif enriched with several positively charged residues, is dispensable for the formation of microtubule–kinetochore attachment and instead functions in silencing the spindle checkpoint^28^. The results present here indicate that Cep57 shows a microtubule-binding mode similar to KNL1, and Cep57 depletion results in no significant defects in microtubule–kinetochore attachment in human cells, which suggests that Cep57 does not play the central role in microtubule–kinetochore attachment, but more likely functions as one of the mediators of checkpoint silencing.

The SAC is associated with mosaic variegated aneuploidy syndrome (MVA), a disease characterized by growth retardation, childhood tumorigenesis, microcephaly and constitutional mosaicism induced by chromosomal gains and losses^62^,^63^. A previous report showed that gene mutations of the spindle checkpoint component BubR1 causes MVA due to defective spindle checkpoint activation^62^. Biallelic loss-of-function *CEP57* mutations also cause MVA, but the molecular mechanism by which such mutations induce aneuploidy syndrome remains obscure^64^. Our findings on the recruitment of Mad1–Mad2 by Cep57 reveal that it functions in the SAC activation and ensures correct chromosome segregation. This may provide the molecular mechanism by which the *CEP57* mutation, similar to BubR1, causes MVA.

## Methods

### Plasmid construction

The full-length complementary DNAs (cDNAs) of Cep57, Mad1, Mad2 and Mis12 were amplified from the HeLa cell cDNA library via PCR, and the primers used are shown in Supplementary Table 1. The full-length and truncated cDNAs were inserted into pEGFP-C2 (Clontech), pEGFP-N3 (Clontech), pET28a (Novagen), pGEX-6P-1 (Amersham Biosciences), pGADT7 (Clontech), pGBKT7 (Clontech), pCMV-myc (Stratagene), p3 × FLAG-CMV-7.1 (Sigma), pHM3 (a gift from Dr Can Xie) and pCMV-tag 2B (Stratagene).

### Antibodies

His-tagged Cep57 protein was injected into mice and rabbits to generate polyclonal antibodies. The anti-Cep57 mouse antibody was used for western blotting (WB, 1:500) and immunofluorescence (IF, 1:50). The anti-Cep57 rabbit antibody was raised and purified using the GST-Cep57 (332–500 amino acids) recombinant protein^34^, and was used for IF (1:10). The other antibodies used for WB or IF were as follows: anti-green fluorescent protein (GFP; clone RQ2, D153-3, MBL) 1:100 WB; anti-HA (clone HA-7, H9658, Sigma) 1:1,000 WB; anti-GAPDH (clone GAPDH-71.1, G9295, Biolinks) 1:1,000 WB; anti-Myc (clone My3, M192-3, MBL) 1:500 WB; anti-Flag (clone M2, F3165, Sigma) 1:1,000 WB, 1:100 IF; anti-α-tubulin (clone DM1A, T9026, Sigma) 1:500 IF; anti-β-tubulin (clone 9F3, 2128, Cell Signaling Technology) 1:500 WB; anti-Cep57 (GTX115931, GeneTex) 1:50 IF; anti-Mad1 (GTX109519, GeneTex) 1:8,000 WB, 1:800 IF; anti-cyclin B1 (clone D5C10, 12231, Cell Signaling Technology) 1:500 WB; anti-securin (ab79546, Abcam) 1:10,000 WB; anti-Mad2 (ab97777, Abcam) 1:500 WB, 1:50 IF; anti-KNL1 (ab70537, Abcam) 1:400 IF; anti-DSN1 (17742-1-AP, Proteintech) 1:200 IF; anti-ZW10 (24561-1-AP, Proteintech) 1:200 IF; anti-Hec1 (clone 9G3.23, GTX70268, GeneTex) 1:500 IF; anti-Zwint-1 (GTX107155, GeneTex) 1:200 IF; anti-CENP-A (clone 3–19, D115-3, MBL) 1:100 IF; anti-Mis12 (ab70843, Abcam) 1:2,000 WB, 1:200 IF, anti-Cdc20 (GTX111137, GeneTex) 1:2,000 WB; anti-BubR1 (11505-2-AP, Proteintech) 1:100 IF; anti-Bub1 (GTX107497, GeneTex) 1:100 IF; anti-CREST (12439-1-AP, gift from Dr Chuanmao Zhang) 1:1,000 IF; anti-GST (sc-33613, Santa Cruz Biotechnology) 1:2,000 WB; and anti-γ-tubulin (T3559 and T6557, Sigma) 1:200 IF. The fluorescent secondary antibodies used were Alexa Fluor 488/568/649-conjugated goat anti-mouse/rabbit/human IgG (Alexa Fluor series, Molecular Probes) 1:200.

### Cell cultures and treatments

HeLa, RPE1 and HEK293T cells were cultured in 10% fetal bovine serum (Gibco) containing DMEM (Gibco) at 37 °C under 5% CO_2_. JetPEI (Polyplus transfection) or Lipofectamine 2000 (Invitrogen) was used to transfect cells according to the manufacturer's instructions.

For the double-thymidine and nocodazole block, HeLa cells were treated with thymidine for 16 h, released for 12 h and were blocked again for 16 h, and then released into nocodazole.

The drugs used were taxol (80 nM, Sigma), MG132 (10 μM, Sigma), nocodazole (100 nM, Invitrogen) and thymidine (2.5 mM, Sigma).

### GST pull-down, immunoprecipitation and western blotting

Cell lysates used for GST pull-down and immunoprecipitation assays were obtained as follows: cells were washed with PBS and then lysed in lysis buffer (150 mM NaCl, 1 mM MgCl_2_, 50 mM Hepes, pH 7.4, 1 mM EGTA and 0.5% Triton X-100) containing protease inhibitors. The lysates were obtained by collecting the supernatant after centrifugation for 10 min at 20,000*g* at 4 °C.

For immunoprecipitation, the cell lysates were incubated with antibodies at 4 °C for 4 h, and were mixed with protein A-Sepharose beads (Amersham Biosciences) for 2 h. The beads were further washed with lysis buffer, and were boiled for 5 min in protein-loading buffer (with SDS).

For GST pull-down assays, the GST and GST-tagged proteins were incubated with Glutathione Sepharose 4B beads (Amersham Bioscience) for purification. Then, the beads were washed five times with lysis buffer, and incubated with the lysates for 4 h at 4 °C. The beads were pelleted and washed five times, and then the sample was boiled with protein-loading buffer (with SDS) for 5 min.

For western blotting, SDS–polyacrylamide gel electrophoresis was used to separate the proteins, and then they were transferred to a polyvinylidene difluoride membrane (Millipore). The membrane was sequentially incubated with primary antibodies and horseradish peroxidase-conjugated protein A (Jackson ImunoResearch) or secondary antibodies. Uncropped scans of typical blots are presented in Supplementary Fig. 8.

### Protein purification and *in vitro* binding assays

Mad1-GFP, Flag-Mad1 and Flag-Cep57 fusion proteins were expressed in HEK293T cells, immunoprecipited with protein A-Sepharose beads (Amersham Biosciences) that were coupled with monoclonal antibodies (anti-GFP, MBL; anti-Flag, Sigma) and then washed five times with lysis buffer containing a high concentration of salt (600 mM NaCl). GST-Mis12, GST-Mad1 (176–718 amino acids), MBP-Cep57 (1–242 amino acids) and MBP-Cep57 (151–500 amino acids) proteins were expressed in *E. coli* (BL21 strain), and were coupled with Glutathione Sepharose 4B beads (Amersham Bioscience) or Amylose Magnetic beads (New England BioLabs) according to the manufacturer's instructions. Then, the beads were washed five times with lysis buffer. For elution, Flag-Cep57 and Flag-Mad1 were eluted by 3 × Flag peptides (Sigma), and GST-tagged fusion proteins were eluted by glutathione (Amersham Bioscience).

For *in vitro* binding assays, ∼0.2 μg of MBP, MBP-Cep57 (1–242 amino acids) and MBP-Cep57 (151–500 amino acids) were coupled with beads and were incubated with ∼0.2 μg of purified and eluted GST-Mis12 or GST-Mad1 (176–718 amino acids), and then the beads were washed five times with lysis buffer. Then, the samples were boiled for 5 min and analysed with western blotting.

### Microtubule polymerization and co-sedimentation assays

Microtubules were assembled at 30 °C for 30 min in BRB80 buffer (100 mM PIPES, 1 mM MgSO_4_, 2 mM EGTA, 1 mM GTP, pH 6.8) stabilized with taxol (20 μM). For microtubule pull-down assays, the taxol-stabilized microtubules were incubated with bead-coupled Cep57 proteins (expressed in bacteria and purified) in BRB80 buffer at room temperature for 1 h, and then the beads were washed three times with BRB80 buffer before boiling. For microtubule co-sedimentation assays, Cep57 expressed in bacteria and purified, and was incubated with assembled microtubules, and the samples were centrifuged at 100,000*g* for 10 min at 25 °C. The supernatant and pellet were boiled and separated by SDS–polyacrylamide gel electrophoresis.

### Immunofluorescence and time-lapse microscopy

Cells were fixed and permeabilized in methanol for 5–10 min at −20 °C, and incubated overnight at 4 °C with primary antibodies in PBS containing 4% bovine serum, followed by staining with secondary antibodies and 1 μg ml^−1^ 4,6-diamidino-2-phenylindole. A confocal microscope (LSM-710 NLO, Zeiss) equipped with a × 100/1.40 numerical aperture (NA) objective lens and a super-resolution confocal microscope (Leica TCS SP8 STED × 3) equipped with a × 100/1.4 NA objective lens were used to observe fixed cells. Three-dimensional super-resolution images were captured using a three-dimensional structured illumination microscope with the N-SIM System (3D-SIM, Nikon). Two time-lapse microscopes, a spinning-disk PerkinElmer UltraView VoX (Nikon) equipped with a × 40/0.9 NA objective lens and an API Delta Vision Elite (Applied Precision) equipped with a × 60/1.40 NA objective lens, were used to visualize the live cells. For time tracking, cells were observed in a chamber at 37 °C under 5% CO_2_ and images were post-processed with Velocity (Nikon) and Delta Vision Softworx (Applied Precision) software.

### RNA interference

The sense-strand sequence of negative control siRNA was 5′- UUCUCCGAACGUGUCACGU -3′. The other siRNA sequences were as follows: Cep57: 5′- AAGCAUGCCGAAAUGGAGAGG -3′, 5′- AACCAUCAAGGUCUAAUGGAA -3′ and 5′- AACCAAAUAACUAAAGUUCGA -3′ (refs 32, 34, 65); Mad1: 5′- CAGGCAGUGUCAGCAGAAC -3′^7^; Mis12: 5′- GGAUCUACAUUGCAUUUCA -3′ and 5′- CCGUUGAACAGGUUAUUCU -3′; Mad2: 5′- GGAAGAGUCGGGACCACAG -3′ (ref. 66). The siRNAs were synthesized by Invitrogen and transfected into cells at 100–120 nM using Lipofectamine 2000 (Invitrogen). For Mad1 depletion, siRNA was transfected twice in 2 days.

The short hairpin RNA (shRNA) vector was pLKO.1-puro (Sigma). The control shRNA sequence was 5′- GCCTTCGTTCATTTACTACTA -3′ and targeting Mad2 shRNA sequences were 5′- CGAGTTCTTCTCATTCGGCAT -3′ and 5′- GCCTTCGTTCATTTACTACTA -3′.

The siRNA-resistant cDNA was cloned by PCR. The siRNA-targeted regions of Cep57 were mutated into 5′- AAACACGCGGAGATGGAAAGA -3′, 5′- AACCTTCTAGCTCAAACGGTA -3′ and 5′- AATCAGATCACCAAGGTCCGG -3′ (ref. 35). The siRNA-targeted regions of Mis12 were mutated into 5′- GGATATATATAGCTTTTCA -3′ and 5′- CCGTAGAGCAAGTAATTCT -3′.

### Yeast two-hybrid assays

The yeast two-hybrid assays were performed according to the Matchmaker Two-Hybrid System Handbook. Mad1, Mad2, Mis12, Cep57 and Cep57R cDNA were amplified from HeLa cell cDNA library and inserted into bait vector pGBKT7 (Clontech Laboratories) and prey vector pGADT7 (Clontech Laboratories). The bait and prey vectors were co-transformed into the AH109 yeast strain, and sequentially plated onto double- (Trp^−^ and Leu^−^) and quadruple- (Trp^−^, Leu^−^, His^−^ and Ade^−^) selective media for 2–5 days at 30 °C.

### Statistical analysis

The fluorescence intensity was measured using Scion Image and Image J software (National Institutes of Health). Statistical analyses were performed using SPSS and GraphPad Prism 5 software. The unpaired two-tailed Student's *t*-test was used to calculate statistical significance.

## Additional information

**How to cite this article:** Zhou, H. *et al*. Cep57 is a Mis12-interacting kinetochore protein involved in kinetochore targeting of Mad1–Mad2. *Nat. Commun.* 7:10151 doi: 10.1038/ncomms10151 (2016).

## Supplementary Material

Supplementary Information Supplementary Figures 1-8 and Supplementary Table 1

Supplementary Movie 1 Control cells arrested in mitosis after nocodazole treatment. HeLa cells were treated with nocodazole after co-transfection with control siRNA and H2B-RFP vector for 48 h. Images were acquired every 30 min. Supplementary movie is presented at 5 fps. Scale bar, 5 μm.

Supplementary Movie 2 Cep57 depleted cells exited from mitosis after nocodazole treatment. HeLa cells were treated with nocodazole after co-transfection with Cep57 siRNA and H2B-RFP vector for 48 h. Images were acquired every 30 min. Supplementary movie is presented at 5 fps. Scale bar, 5 μm.

Supplementary Movie 3 Control RPE1 cells showed normal chromosome segregation. Cells were co-transfected with control siRNA and H2B-GFP vector for 60 h. Images were acquired every 3 min. Supplementary movie is presented at 5 fps. Scale bar, 5 μm.

Supplementary Movie 4 Cep57 depleted RPE1 cells showed immature anaphase onset and chromosome lagging. Cells were co-transfected with Cep57 siRNA and H2B-GFP vector for 60 h. Images were acquired every 3 min. Supplementary movie is presented at 5 fps. Scale bar, 5 μm.

Supplementary Movie 5 Control HeLa cells showed normal chromosome segregation. Cells were co-transfected with control siRNA and H2B-RFP vector for 60 h. Images were acquired every 3 min. Supplementary movie is presented at 5 fps. Scale bar, 5 μm.

Supplementary Movie 6 Cep57 depleted HeLa cells showed immature anaphase onset and chromosome lagging. Cells were co-transfected with Cep57 siRNA and H2B-RFP vector for 60 h. Images were acquired every 3 min. Supplementary movie is presented at 5 fps. Scale bar, 5 μm.

## References

[b1] Foley E. A. & Kapoor T. M. Microtubule attachment and spindle assembly checkpoint signalling at the kinetochore. Nat. Rev. Mol. Cell Biol. 14, 25–37 (2013) . 23258294 10.1038/nrm3494 PMC3762224

[b2] Musacchio A. & Salmon E. D. The spindle-assembly checkpoint in space and time. Nat. Rev. Mol. Cell Biol. 8, 379–393 (2007) . 17426725 10.1038/nrm2163

[b3] Chen R. H., Shevchenko A., Mann M. & Murray A. W. Spindle checkpoint protein Xmad1 recruits Xmad2 to unattached kinetochores. J. Cell Biol. 143, 283–295 (1998) . 9786942 10.1083/jcb.143.2.283 PMC2132829

[b4] Fava L. L., Kaulich M., Nigg E. A. & Santamaria A. Probing the in vivo function of Mad1:C-Mad2 in the spindle assembly checkpoint. EMBO J. 30, 3322–3336 (2011) . 21772247 10.1038/emboj.2011.239 PMC3160659

[b5] Sironi L. . Mad2 binding to Mad1 and Cdc20, rather than oligomerization, is required for the spindle checkpoint. EMBO J. 20, 6371–6382 (2001) . 11707408 10.1093/emboj/20.22.6371 PMC125308

[b6] De Antoni A. . The Mad1/Mad2 complex as a template for Mad2 activation in the spindle assembly checkpoint. Curr. Biol. 15, 214–225 (2005) . 15694304 10.1016/j.cub.2005.01.038

[b7] Luo X., Tang Z., Rizo J. & Yu H. The Mad2 spindle checkpoint protein undergoes similar major conformational changes upon binding to either Mad1 or Cdc20. Mol. Cell 9, 59–71 (2002) . 11804586 10.1016/s1097-2765(01)00435-x

[b8] Kulukian A., Han J. S. & Cleveland D. W. Unattached kinetochores catalyze production of an anaphase inhibitor that requires a Mad2 template to prime Cdc20 for BubR1 binding. Dev. Cell 16, 105–117 (2009) . 19154722 10.1016/j.devcel.2008.11.005 PMC2655205

[b9] Malureanu L. A. . BubR1 N terminus acts as a soluble inhibitor of cyclin B degradation by APC/C(Cdc20) in interphase. Dev. Cell 16, 118–131 (2009) . 19154723 10.1016/j.devcel.2008.11.004 PMC2659634

[b10] Sudakin V., Chan G. K. & Yen T. J. Checkpoint inhibition of the APC/C in HeLa cells is mediated by a complex of BUBR1, BUB3, CDC20, and MAD2. J. Cell Biol. 154, 925–936 (2001) . 11535616 10.1083/jcb.200102093 PMC2196190

[b11] Tang Z., Bharadwaj R., Li B. & Yu H. Mad2-Independent inhibition of APCCdc20 by the mitotic checkpoint protein BubR1. Dev. Cell 1, 227–237 (2001) . 11702782 10.1016/s1534-5807(01)00019-3

[b12] Yu H. Regulation of APC-Cdc20 by the spindle checkpoint. Curr. Opin. Cell Biol. 14, 706–714 (2002) . 12473343 10.1016/s0955-0674(02)00382-4

[b13] Musacchio A. Spindle assembly checkpoint: the third decade. Philos. Trans. R. Soc. Lond. B Biol. Sci. 366, 3595–3604 (2011) . 22084386 10.1098/rstb.2011.0072 PMC3203455

[b14] DeLuca J. G. . Nuf2 and Hec1 are required for retention of the checkpoint proteins Mad1 and Mad2 to kinetochores. Curr. Biol. 13, 2103–2109 (2003) . 14654001 10.1016/j.cub.2003.10.056

[b15] Karess R. Rod-Zw10-Zwilch: a key player in the spindle checkpoint. Trends Cell Biol. 15, 386–392 (2005) . 15922598 10.1016/j.tcb.2005.05.003

[b16] Sharp-Baker H. & Chen R. H. Spindle checkpoint protein Bub1 is required for kinetochore localization of Mad1, Mad2, Bub3, and CENP-E, independently of its kinase activity. J. Cell Biol. 153, 1239–1250 (2001) . 11402067 10.1083/jcb.153.6.1239 PMC2192030

[b17] Chan G. K., Jablonski S. A., Starr D. A., Goldberg M. L. & Yen T. J. Human Zw10 and ROD are mitotic checkpoint proteins that bind to kinetochores. Nat. Cell Biol. 2, 944–947 (2000) . 11146660 10.1038/35046598

[b18] Liu S. T. . Human CENP-I specifies localization of CENP-F, MAD1 and MAD2 to kinetochores and is essential for mitosis. Nat. Cell Biol. 5, 341–345 (2003) . 12640463 10.1038/ncb953

[b19] Kops G. J. . ZW10 links mitotic checkpoint signaling to the structural kinetochore. J. Cell Biol. 169, 49–60 (2005) . 15824131 10.1083/jcb.200411118 PMC1351127

[b20] Buffin E., Lefebvre C., Huang J., Gagou M. E. & Karess R. E. Recruitment of Mad2 to the kinetochore requires the Rod/Zw10 complex. Curr. Biol. 15, 856–861 (2005) . 15886105 10.1016/j.cub.2005.03.052

[b21] Lara-Gonzalez P., Westhorpe F. G. & Taylor S. S. The spindle assembly checkpoint. Curr. Biol. 22, R966–R980 (2012) . 23174302 10.1016/j.cub.2012.10.006

[b22] Wei R., Ngo B., Wu G. & Lee W. H. Phosphorylation of the Ndc80 complex protein, HEC1, by Nek2 kinase modulates chromosome alignment and signaling of the spindle assembly checkpoint. Mol. Biol. Cell 22, 3584–3594 (2011) . 21832156 10.1091/mbc.E11-01-0012 PMC3183014

[b23] Martin-Lluesma S., Stucke V. M. & Nigg E. A. Role of Hec1 in spindle checkpoint signaling and kinetochore recruitment of Mad1/Mad2. Science 297, 2267–2270 (2002) . 12351790 10.1126/science.1075596

[b24] Moyle M. W. . A Bub1-Mad1 interaction targets the Mad1-Mad2 complex to unattached kinetochores to initiate the spindle checkpoint. J. Cell Biol. 204, 647–657 (2014) . 24567362 10.1083/jcb.201311015 PMC3941058

[b25] London N. & Biggins S. Mad1 kinetochore recruitment by Mps1-mediated phosphorylation of Bub1 signals the spindle checkpoint. Genes Dev. 28, 140–152 (2014) . 24402315 10.1101/gad.233700.113 PMC3909788

[b26] London N., Ceto S., Ranish J. A. & Biggins S. Phosphoregulation of Spc105 by Mps1 and PP1 regulates Bub1 localization to kinetochores. Curr. Biol. 22, 900–906 (2012) . 22521787 10.1016/j.cub.2012.03.052 PMC3723133

[b27] Yamagishi Y., Yang C. H., Tanno Y. & Watanabe Y. MPS1/Mph1 phosphorylates the kinetochore protein KNL1/Spc7 to recruit SAC components. Nat. Cell Biol. 14, 746–752 (2012) . 22660415 10.1038/ncb2515

[b28] Espeut J., Cheerambathur D. K., Krenning L., Oegema K. & Desai A. Microtubule binding by KNL-1 contributes to spindle checkpoint silencing at the kinetochore. J. Cell Biol. 196, 469–482 (2012) . 22331849 10.1083/jcb.201111107 PMC3284002

[b29] Krenn V., Wehenkel A., Li X., Santaguida S. & Musacchio A. Structural analysis reveals features of the spindle checkpoint kinase Bub1-kinetochore subunit Knl1 interaction. J. Cell Biol. 196, 451–467 (2012) . 22331848 10.1083/jcb.201110013 PMC3283998

[b30] Burke D. J. & Stukenberg P. T. Linking kinetochore-microtubule binding to the spindle checkpoint. Dev. Cell 14, 474–479 (2008) . 18410725 10.1016/j.devcel.2008.03.015 PMC2696048

[b31] Emanuele M. J. & Stukenberg P. T. Xenopus Cep57 is a novel kinetochore component involved in microtubule attachment. Cell 130, 893–905 (2007) . 17803911 10.1016/j.cell.2007.07.023

[b32] Bossard C. . Translokin is an intracellular mediator of FGF-2 trafficking. Nat. Cell Biol. 5, 433–439 (2003) . 12717444 10.1038/ncb979

[b33] Momotani K., Khromov A. S., Miyake T., Stukenberg P. T. & Somlyo A. V. Cep57, a multidomain protein with unique microtubule and centrosomal localization domains. Biochem. J. 412, 265–273 (2008) . 18294141 10.1042/BJ20071501 PMC4351815

[b34] Wu Q. . Cep57, a NEDD1-binding pericentriolar material component, is essential for spindle pole integrity. Cell Res. 22, 1390–1401 (2012) . 22508265 10.1038/cr.2012.61 PMC3434346

[b35] He R. . Cep57 protein is required for cytokinesis by facilitating central spindle microtubule organization. J. Biol. Chem. 288, 14384–14390 (2013) . 23569207 10.1074/jbc.M112.441501 PMC3656293

[b36] Lukinavicius G. . Selective chemical crosslinking reveals a Cep57-Cep63-Cep152 centrosomal complex. Curr. Biol. 23, 265–270 (2013) . 23333316 10.1016/j.cub.2012.12.030

[b37] Cuevas R., Korzeniewski N., Tolstov Y., Hohenfellner M. & Duensing S. FGF-2 disrupts mitotic stability in prostate cancer through the intracellular trafficking protein CEP57. Cancer Res. 73, 1400–1410 (2013) . 23243019 10.1158/0008-5472.CAN-12-1857

[b38] Fritzler M. J. & Kinsella T. D. The CREST syndrome: a distinct serologic entity with anticentromere antibodies. Am. J. Med. 69, 520–526 (1980) . 6968511 10.1016/0002-9343(80)90462-3

[b39] Warburton P. E. . Immunolocalization of CENP-A suggests a distinct nucleosome structure at the inner kinetochore plate of active centromeres. Curr. Biol. 7, 901–904 (1997) . 9382805 10.1016/s0960-9822(06)00382-4

[b40] Petrovic A. . The MIS12 complex is a protein interaction hub for outer kinetochore assembly. J. Cell Biol. 190, 835–852 (2010) . 20819937 10.1083/jcb.201002070 PMC2935574

[b41] Obuse C. . A conserved Mis12 centromere complex is linked to heterochromatic HP1 and outer kinetochore protein Zwint-1. Nat. Cell Biol. 6, 1135–1141 (2004) . 15502821 10.1038/ncb1187

[b42] Varma D. . Spindle assembly checkpoint proteins are positioned close to core microtubule attachment sites at kinetochores. J. Cell Biol. 202, 735–746 (2013) . 23979716 10.1083/jcb.201304197 PMC3760617

[b43] Famulski J. K., Vos L., Sun X. & Chan G. Stable hZW10 kinetochore residency, mediated by hZwint-1 interaction, is essential for the mitotic checkpoint. J. Cell Biol. 180, 507–520 (2008) . 18268100 10.1083/jcb.200708021 PMC2234252

[b44] Ohta S. . The protein composition of mitotic chromosomes determined using multiclassifier combinatorial proteomics. Cell 142, 810–821 (2010) . 20813266 10.1016/j.cell.2010.07.047 PMC2982257

[b45] Liu S. T., Rattner J. B., Jablonski S. A. & Yen T. J. Mapping the assembly pathways that specify formation of the trilaminar kinetochore plates in human cells. J. Cell Biol. 175, 41–53 (2006) . 17030981 10.1083/jcb.200606020 PMC2064494

[b46] McCleland M. L. . The highly conserved Ndc80 complex is required for kinetochore assembly, chromosome congression, and spindle checkpoint activity. Genes Dev. 17, 101–114 (2003) . 12514103 10.1101/gad.1040903 PMC195965

[b47] Wang H. . Human Zwint-1 specifies localization of Zeste White 10 to kinetochores and is essential for mitotic checkpoint signaling. J. Biol. Chem. 279, 54590–54598 (2004) . 15485811 10.1074/jbc.M407588200

[b48] Kim S., Sun H., Tomchick D. R., Yu H. & Luo X. Structure of human Mad1 C-terminal domain reveals its involvement in kinetochore targeting. Proc. Natl Acad. Sci. USA 109, 6549–6554 (2012) . 22493223 10.1073/pnas.1118210109 PMC3340083

[b49] Mapelli M., Massimiliano L., Santaguida S. & Musacchio A. The Mad2 conformational dimer: structure and implications for the spindle assembly checkpoint. Cell 131, 730–743 (2007) . 18022367 10.1016/j.cell.2007.08.049

[b50] Kim S. . Phosphorylation of the spindle checkpoint protein Mad2 regulates its conformational transition. Proc. Natl Acad. Sci. USA 107, 19772–19777 (2010) . 21041666 10.1073/pnas.1009000107 PMC2993392

[b51] Chan Y. W. . Mitotic control of kinetochore-associated dynein and spindle orientation by human Spindly. J. Cell Biol. 185, 859–874 (2009) . 19468067 10.1083/jcb.200812167 PMC2711594

[b52] Wojcik E. . Kinetochore dynein: its dynamics and role in the transport of the Rough deal checkpoint protein. Nat. Cell Biol. 3, 1001–1007 (2001) . 11715021 10.1038/ncb1101-1001

[b53] Howell B. J. . Cytoplasmic dynein/dynactin drives kinetochore protein transport to the spindle poles and has a role in mitotic spindle checkpoint inactivation. J. Cell Biol. 155, 1159–1172 (2001) . 11756470 10.1083/jcb.200105093 PMC2199338

[b54] Griffis E. R., Stuurman N. & Vale R. D. Spindly, a novel protein essential for silencing the spindle assembly checkpoint, recruits dynein to the kinetochore. J. Cell Biol. 177, 1005–1015 (2007) . 17576797 10.1083/jcb.200702062 PMC2064361

[b55] Gassmann R. . Removal of Spindly from microtubule-attached kinetochores controls spindle checkpoint silencing in human cells. Genes Dev. 24, 957–971 (2010) . 20439434 10.1101/gad.1886810 PMC2861194

[b56] Barisic M. . Spindly/CCDC99 is required for efficient chromosome congression and mitotic checkpoint regulation. Mol. Biol. Cell 21, 1968–1981 (2010) . 20427577 10.1091/mbc.E09-04-0356 PMC2883941

[b57] Waters J. C., Chen R. H., Murray A. W. & Salmon E. D. Localization of Mad2 to kinetochores depends on microtubule attachment, not tension. J. Cell Biol. 141, 1181–1191 (1998) . 9606210 10.1083/jcb.141.5.1181 PMC2137189

[b58] Rosenberg J. S., Cross F. R. & Funabiki H. KNL1/Spc105 recruits PP1 to silence the spindle assembly checkpoint. Curr. Biol. 21, 942–947 (2011) . 21640906 10.1016/j.cub.2011.04.011 PMC3109435

[b59] Ji Z., Gao H. & Yu H. CELL DIVISION CYCLE. Kinetochore attachment sensed by competitive Mps1 and microtubule binding to Ndc80C. Science 348, 1260–1264 (2015) . 26068854 10.1126/science.aaa4029

[b60] Hiruma Y. . CELL DIVISION CYCLE. Competition between MPS1 and microtubules at kinetochores regulates spindle checkpoint signaling. Science 348, 1264–1267 (2015) . 26068855 10.1126/science.aaa4055

[b61] Cheeseman I. M. & Desai A. Molecular architecture of the kinetochore-microtubule interface. Nat. Rev. Mol. Cell Biol. 9, 33–46 (2008) . 18097444 10.1038/nrm2310

[b62] Hanks S. . Constitutional aneuploidy and cancer predisposition caused by biallelic mutations in BUB1B. Nat. Genet. 36, 1159–1161 (2004) . 15475955 10.1038/ng1449

[b63] Suijkerbuijk S. J. . Molecular causes for BUBR1 dysfunction in the human cancer predisposition syndrome mosaic variegated aneuploidy. Cancer Res. 70, 4891–4900 (2010) . 20516114 10.1158/0008-5472.CAN-09-4319 PMC2887387

[b64] Snape K. . Mutations in CEP57 cause mosaic variegated aneuploidy syndrome. Nat. Genet. 43, 527–529 (2011) . 21552266 10.1038/ng.822 PMC3508359

[b65] Graser S. . Cep164, a novel centriole appendage protein required for primary cilium formation. J. Cell Biol. 179, 321–330 (2007) . 17954613 10.1083/jcb.200707181 PMC2064767

[b66] Lischetti T., Zhang G., Sedgwick G. G., Bolanos-Garcia V. M. & Nilsson J. The internal Cdc20 binding site in BubR1 facilitates both spindle assembly checkpoint signalling and silencing. Nat. Commun. 5, 5563 (2014) . 25482201 10.1038/ncomms6563

