# Supplementary material for: Cep57 is a Mis12-interacting kinetochore protein involved in kinetochore targeting of Mad1–Mad2
Source: Nat Commun. 2016 Jan 8;7:10151. doi: 10.1038/ncomms10151 (PMC4729865; doi:10.1038/ncomms10151)
Supplement: Supplementary Information — Supplementary Figures 1-8 and Supplementary Table 1 [file ncomms10151-s1.pdf]

Supplementary Figures and Legends

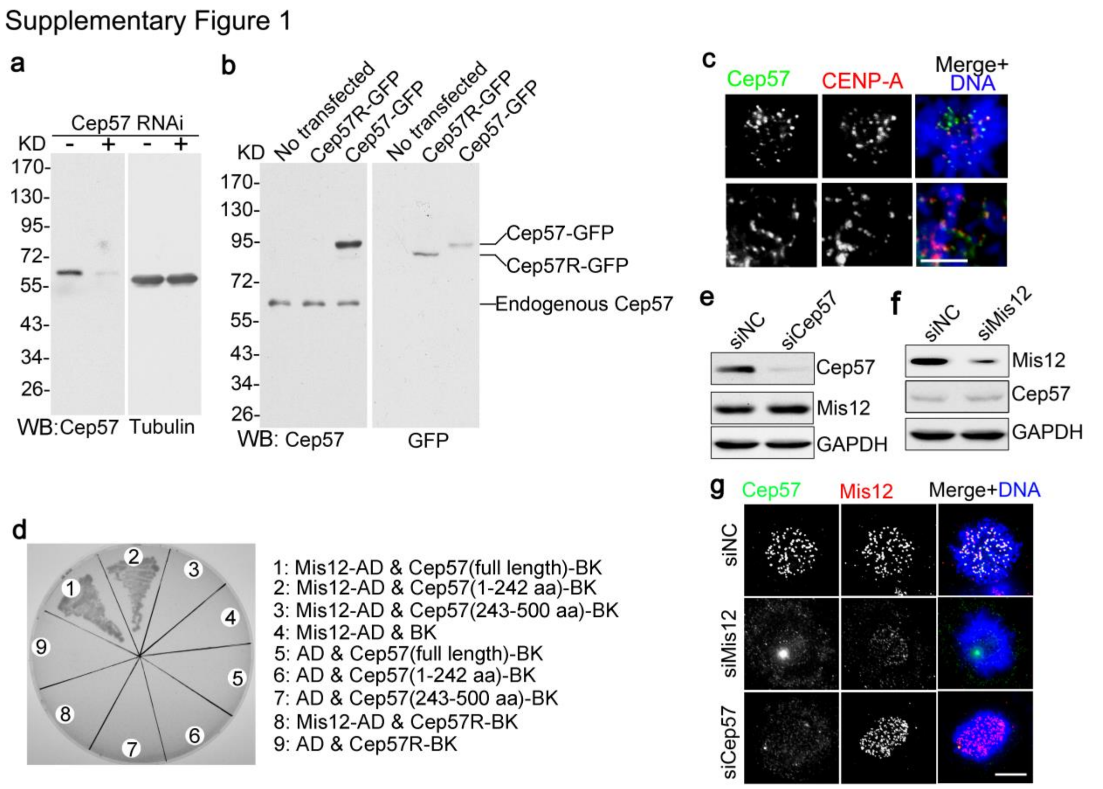

Supplementary Figure 1. Cep57 is a kinetochore-localized protein. (**a**) Western blots (WB) showing Cep57 protein levels in control (–) and Cep57 siRNA-treated cells (+) with anti-Cep57 mouse polyclonal antibody (left). Tubulin served as a loading control (right). (**b**) Western blots (WB) showing Cep57R-GFP and Cep57-GFP overexpressed in HeLa cells, and endogenous Cep57 with anti-Cep57 and anti-GFP antibodies. (**c**) Immunostaining of Cep57 (green) and CENP-A (red) in HeLa cells with anti-CENP-A antibody and anti-Cep57 rabbit polyclonal antibodies (GTX115931, GeneTex, top; Lab raised antibody, bottom). DNA was stained with DAPI (blue). Scale bar, 5 μm. (**d**) Yeast two-hybrid assays. The yeasts transformed with the indicated plasmids were plated onto quadruple selective medium (Trp$^-$, Leu$^-$, His$^-$, Ade$^-$) for 2 days at 30°C. AD, pGADT7; BK, pGBKT7. (**e**,**f**) Western blotting of the indicated proteins in HeLa cells depleted of negative control (NC), Cep57 (60 h) (**e**), or Mis12 (85 h) (**f**) by siRNAs.

GAPDH served as a loading control. (**g**) Immunostaining of Cep57 (green) and Mis12 (red) in HeLa cells depleted of negative control (NC), Cep57, or Mis12 by siRNA. DNA was stained with DAPI (blue). Scale bar, 5 μm.

Supplementary Figure 2

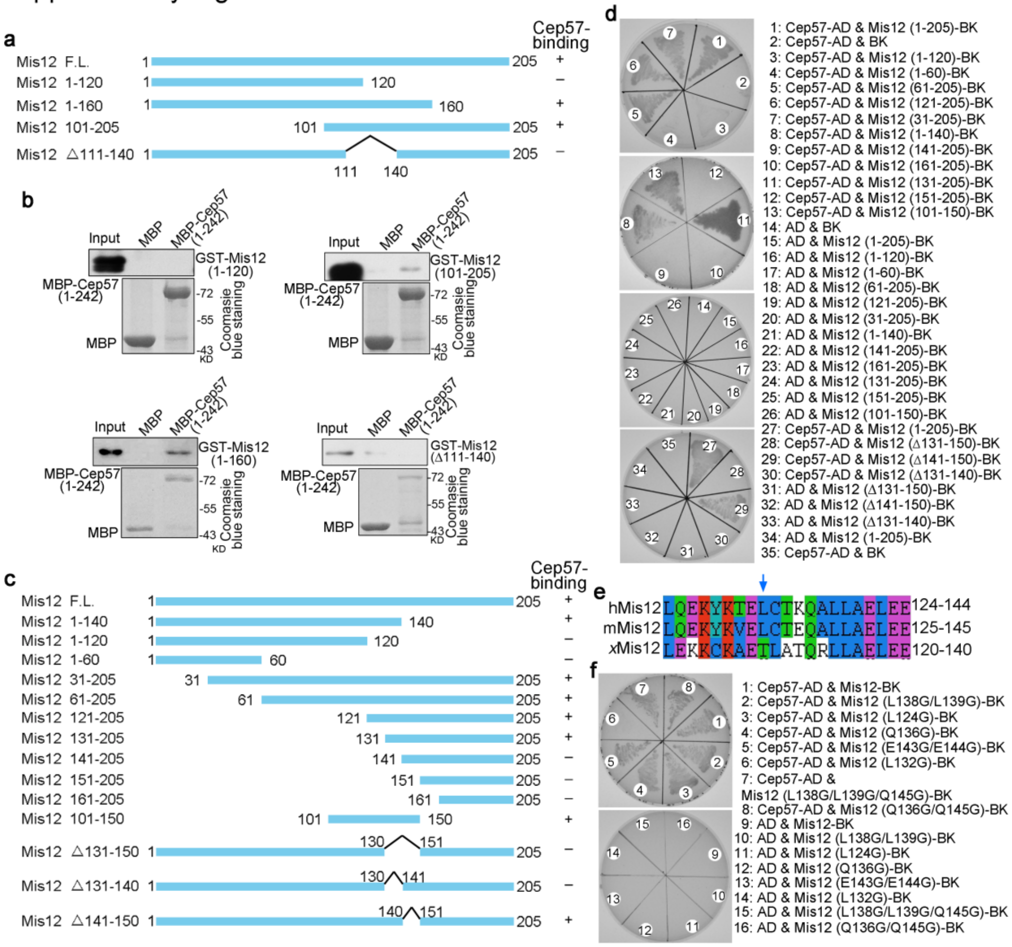

Supplementary Figure 2. The 131-140 aa region of Mis12 is critical for its binding with Cep57. (**a**, **c**) Schematic of Mis12 mutants used for pull-down assays (**a**) and yeast two-hybrid assays (**c**). (**b**) *In vitro* pull-down assays. GST-tagged mutants of Mis12 and MBP-Cep57 (1-242 aa) expressed in *E. coli* and purified were incubated with Amylose Magnetic beads. The precipitated samples were analyzed by western blotting with anti-GST antibody (GST-Mis12) and Coomassie blue staining (MBP and MBP-Cep57). (**d**) Yeast two-hybrid assays. The yeasts transformed with the indicated plasmids were plated onto quadruple selective medium (Trp$^-$, Leu$^-$, His$^-$, Ade$^-$) for 2 days at 30°C. AD, pGADT7; BK, pGBKT7. (**e**) Alignment of Mis12 amino-acids sequences from human (h), mouse (m), and *Xenopus* (*x*). (**f**) Yeast two-hybrid assays. The yeasts transformed with the indicated plasmids were plated onto quadruple selective medium (Trp$^-$, Leu$^-$,

His^{-}, Ade^{-}) for 2 days at 30°C. AD, pGADT7; BK, pGBKT7.

Supplementary Figure 3

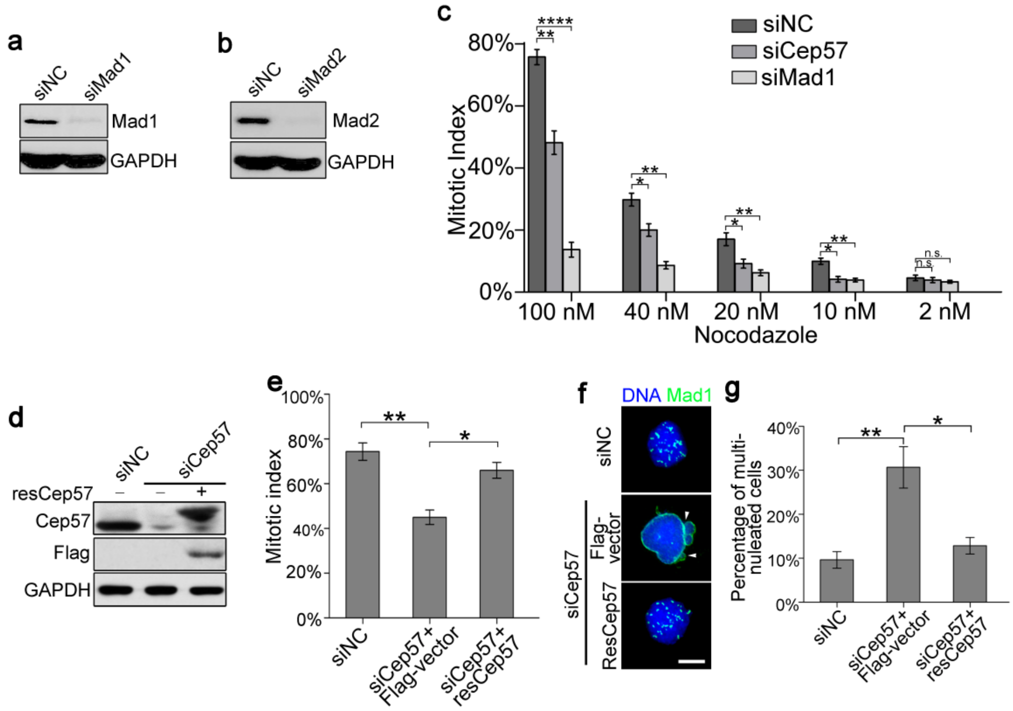

Supplementary Figure 3. Cep57 depletion weakens activation of spindle assembly checkpoint. (**a**,**b**) Western blots show the significant decrease of Mad1 (**a**) and Mad2 (**b**) proteins by siRNA for 48 h in HeLa cells. GAPDH served as a loading control. (**c**) Quantification of the percentage of mitotic HeLa cells after depletion of Cep57 or Mad1 by siRNA for 48 h followed by treatment with the indicated concentration of nocodazole for 18 h. NC, negative control. The experiment was repeated three times. (**d-g**) Depletion of Cep57 by siRNA, and rescued by expressing siRNA-resistant Flag-tagged Cep57 (resCep57) for 48 h in HeLa cells. NC, negative control. (**d**) Western blots show the significant decrease of Cep57 protein level by siRNA transfection, and rescued by overexpressing Flag-tagged resCep57. (**e**) Quantification of the percentage of mitotic cells after treatment with 100 nM nocodazole for 18 h. *n*>300 cells per experiment. The experiment was repeated three times. (**f**) Immunofluorescence of Mad1 (green) in HeLa cells. DNA was stained with DAPI (blue). Arrowheads, multi-nuclei. Scale bar, 5 μm. (**g**) Quantification of the percentage of cells with multi-nuclei in (**f**). *n*>300 cells per experiment. The

experiment was repeated three times. For (**c**), (**e**) and (**g**), data are mean ± s.e.m.. ****$P<0.0001$; **$P<0.01$; *$P<0.05$; n.s., not significant (unpaired two-tailed Student's *t*-test).

Supplementary Figure 4

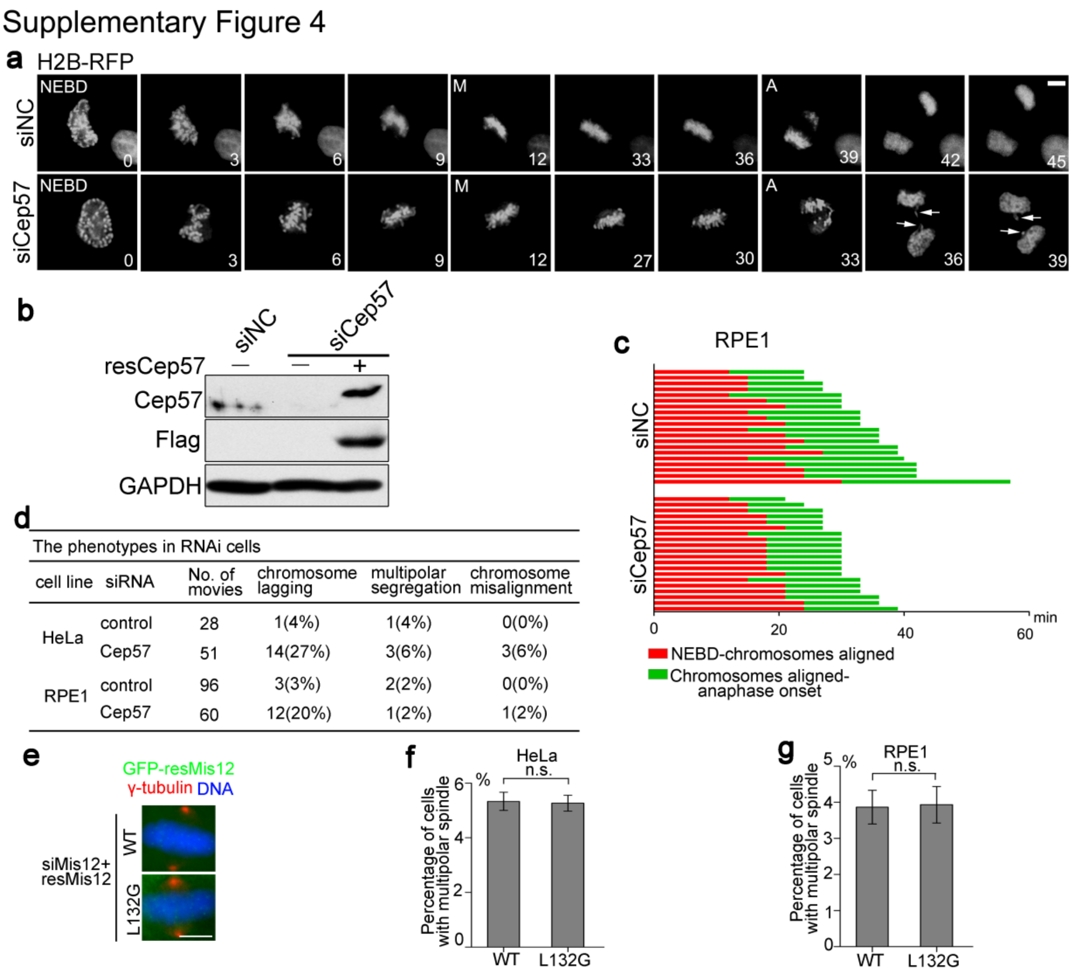

d

The phenotypes in RNAi cells

cell line	siRNA	No. of movies	chromosome lagging	multipolar segregation	chromosome misalignment
HeLa	control	28	1(4%)	1(4%)	0(0%)
	Cep57	51	14(27%)	3(6%)	3(6%)
RPE1	control	96	3(3%)	2(2%)	0(0%)
	Cep57	60	12(20%)	1(2%)	1(2%)

Supplementary Figure 4. Cep57 depletion accelerates anaphase onset and leads to chromosome lagging. (**a**) Time-lapse images of HeLa cells co-transfecting H2B-RFP and negative control (NC)- or Cep57-siRNA for 60 h. NEBD, nuclear envelope breakdown. M, metaphase; A, anaphase. The numbers indicate the time (minutes) after NEBD. Arrows, lagging chromosome. Scale bar, 5 μm. (**b**) Western blots show the significant decrease of Cep57 protein level by siRNA in RPE1 cells, and rescued by expressing siRNA-resistant Flag-tagged Cep57 (resCep57). GAPDH served as a loading control. (**c**) Mitotic progression from NEBD to anaphase onset of RPE1 cells co-transfected with H2B-GFP and negative control (NC)- or Cep57-siRNA for 60 h. The cells were selected at random. (**d**) The frequency of cells showing chromosome lagging

and other phenotypes from (**a**) and Figure 4**a**. (**e**) Immunofluorescence of γ-tubulin (red) in HeLa cells transfected with Mis12 siRNA together with siRNA-resistant GFP-Mis12 (resMis12). DNA was stained with DAPI (blue). Scale bar, 5 μm. (**f,g**) Quantification of the percentage of cells with multipolar spindles in HeLa cells (**f**) and RPE1 cells (**g**) transfected with Mis12 siRNA together with siRNA-resistant GFP-Mis12 (resMis12). The experiment was repeated three times. For (**f**) and (**g**), data are mean ± s.e.m.. n.s., not significant (unpaired two-tailed Student's *t*-test).

Supplementary Figure 5

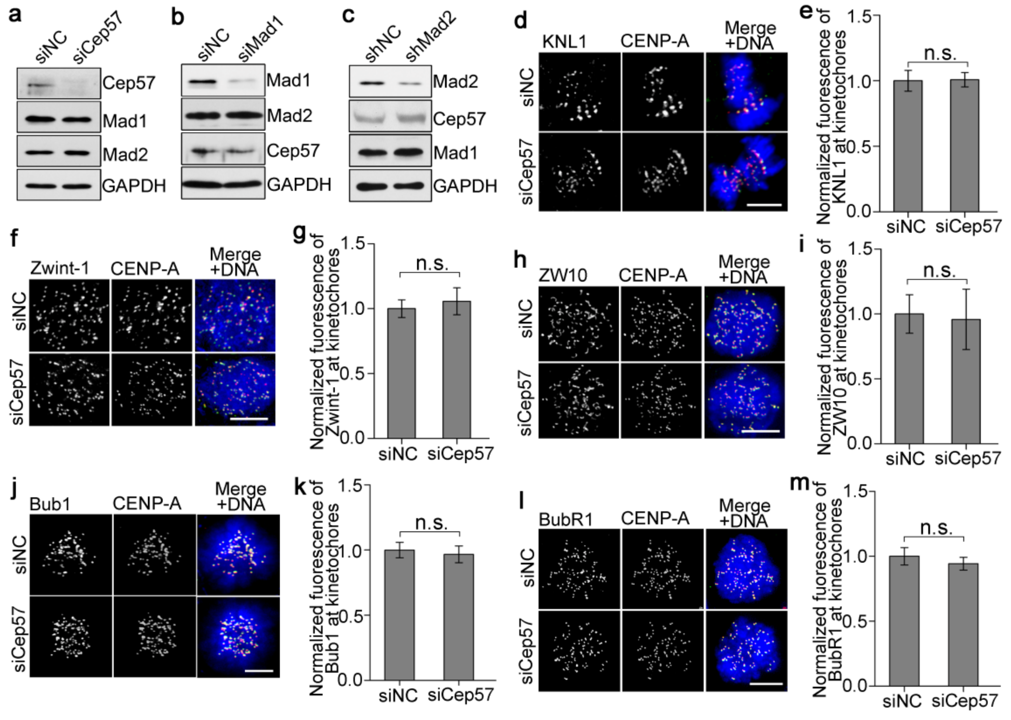

Supplementary Figure 5. Cep57 does not affect the kinetochore localization of KNL1, Zwint-1, ZW10, Bub1, or BubR1. (**a**,**b**) Depletion of Cep57 (**a**) or Mad1 (**b**) in HeLa cells by siRNA for 60 h. The indicated proteins were detected by western blotting. GAPDH served as a loading control. NC, negative control. (**c**) HeLa cells were transfected with plasmids expressing negative control (NC)- or Mad2-shRNA for 48 h. The indicated proteins were detected by western blotting. (**d**,**f**,**h**,**j**,**l**) Immunostaining of CENP-A (red) together with KNL1 (green) (**d**), Zwint-1 (green) (**f**), ZW10 (green) (**h**), Bub1 (green) (**j**), or BubR1 (green) (**l**) in HeLa cells after transfected with negative control (NC)- or Cep57-siRNA for 48 h and treatment with (**f**,**h**,**j**,**l**) or without nocodazole and MG132 for 1 h (**d**). DNA was stained with DAPI (blue). Scale bars, 5 μm. (**e**,**g**,**i**,**k**,**m**) Quantification and normalization of the kinetochore signal of KNL1 (**e**) from (**d**), Zwint-1 (**g**) from (**f**), ZW10 (**i**) from (**h**), Bub1 (**k**) from (**j**), and BubR1 (**m**) from (**l**). >200 kinetochores from 20 cells were measured. The experiment was repeated three times. Data are mean ± s.e.m.. n.s., not significant (unpaired two-tailed Student's *t*-test).

Supplementary Figure 6

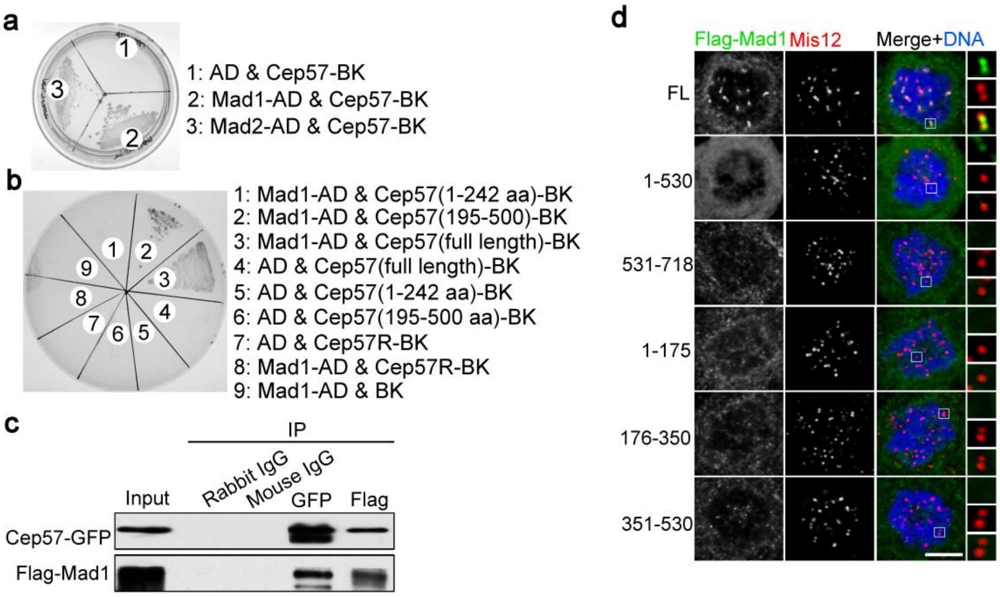

Supplementary Figure 6. The structural integrity of Mad1 is essential for its kinetochore targeting. (**a,b**) Yeast two-hybrid assays. The yeasts transformed with the indicated plasmids were plated onto quadruple selective medium (Trp$^-$, Leu$^-$, His$^-$, Ade$^-$) for 3 days at 30°C. AD, pGADT7; BK, pGBKT7. (**c**) HEK293T cells were co-transfected with Cep57-GFP and Flag-Mad1, and were used to perform immunoprecipitation (IP) and western blotting with anti-GFP and anti-Flag antibodies. (**d**) Full-length and truncated mutants of Flag-Mad1 were overexpressed in HeLa cells, and the cells were treated with nocodazole for 1 h and immunostained for Flag-Mad1 (green) and Mis12 (red). DNA was stained with DAPI (blue). Scale bar, 5 μm.

Supplementary Figure 7

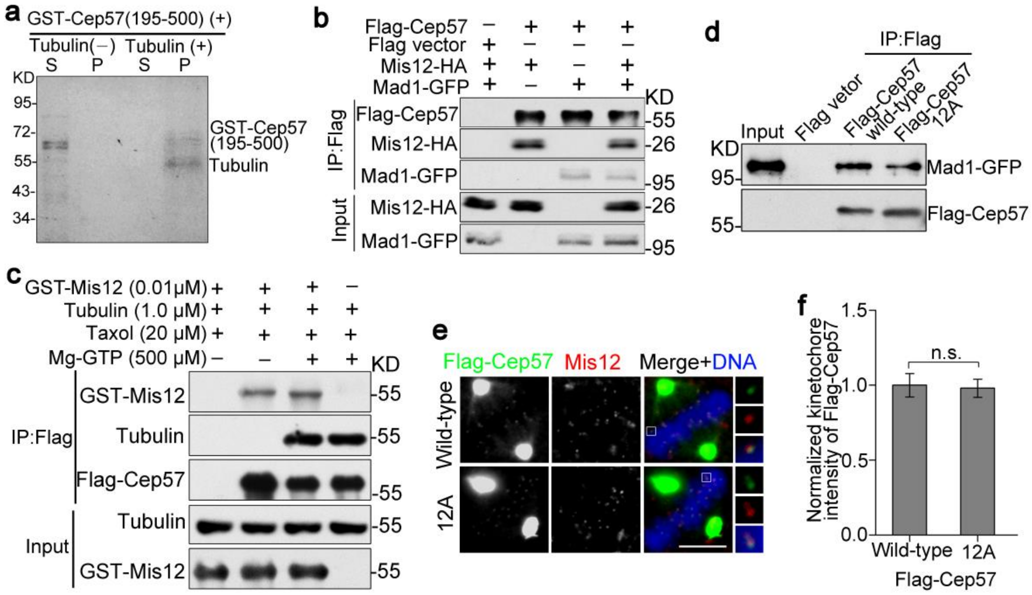

Supplementary Figure 7. Cep57 binds to microtubule, Mis12 and Mad1. (**a**) Microtubule-binding assays *in vitro*. Cep57 (195-500 aa) (0.1 μM) expressed and purified from *E. coli* was incubated with or without taxol-stabilized microtubules (0.2 μM) in BRB80 buffer. After centrifugation, supernatant (S) and pellet (P) were separated and analyzed with SDS-PAGE and Coomassie blue staining. (**b**) HEK293T cells were co-transfected with the indicated plasmids, and were used to perform immunoprecipitation (IP) and western blotting for detecting the indicated proteins. (**c**) Tubulin in BRB80 buffer with or without GTP was incubated with GST-Mis12 (expressed in *E. coli* and purified) and protein A-Sepharose beads coupled Flag-Cep57 (expressed in HEK293T cells and purified) with anti-Flag antibody at room temperature. The beads associated proteins were analyzed by western blotting for detecting the indicated proteins. (**d**) Mad1-GFP and wild-type Flag-Cep57 or mutant Flag-Cep57-12A expressed in HEK293T cells were used to perform IP and western blotting with anti-GFP and anti-Flag antibodies. 12A: K432A, K434A, K435A, K438A, K441A, K442A, K467A, R469A, K473A, R474A, R475A, and K476A. (**e**) Metaphase HeLa cells expressing RNAi-resistant Flag-Cep57 or Flag-Cep57-12A

after treatment with Cep57 RNAi were immunostained for Flag-Cep57 (green) and Mis12 (red). DNA was stained with DAPI (blue). Scale bar, 5 μm. (**f**) Quantification and normalization of the kinetochore signal of Flag-Cep57 from (**e**). >100 kinetochores from 10 cells were measured. The experiment was repeated three times. Data are mean ± s.e.m.. n.s., not significant (unpaired two-tailed Student's *t*-test).

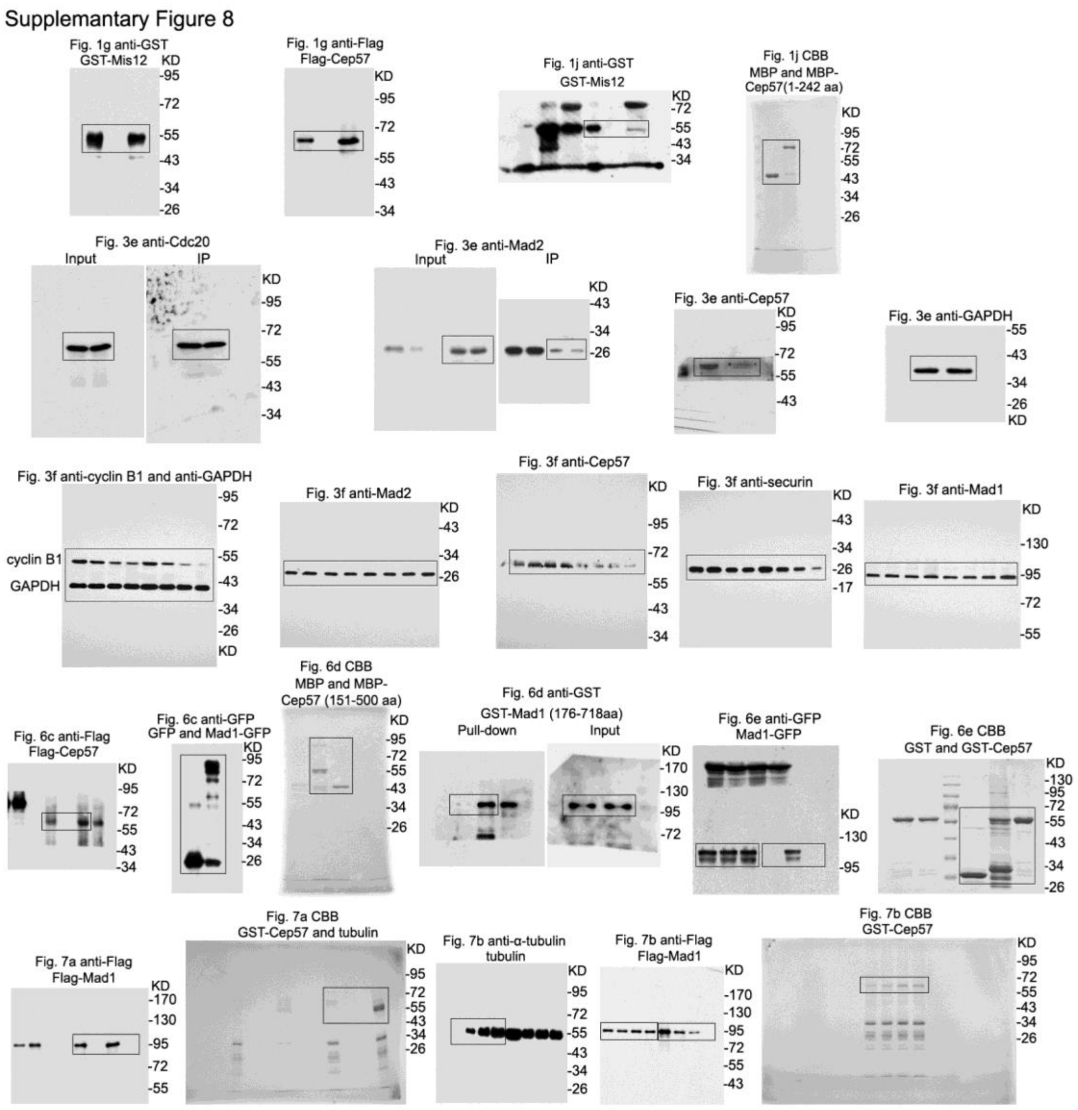

Supplementary Figure 8. Images of uncropped scans of representative immunoblots and CBB gels. Black boxes indicate the cropped regions that are shown in the indicated figures. CBB, Coomassie blue staining.

Supplementary Table 1. Primers used to amplifying full-length cDNAs of Cep57, Mis12, Mad1, and Mad2.

Primer name	Primer sequence
hCep57-F	5’-AGAGAGAGAGATCTATGGCGGCGGCGTCTGTCTC-3’
hCep57-R	5’-GAGAGAGAGTCGACGTAATCCCAACACAAACTACT-3’
Mad2 humanF	5’-AGCGAATTCATGGCGCTGCAGCTCTCCC-3’
Mad2 humanR	5’-AGCGTCGACGTCATTGACAGGAATTTTGTAG-3’
Mad1-R-BamHI	5’-GAGA GGATCCCGCCACGGTCTGGCGGCTG-3’
Mad1-F-ECORI	5’-GAGAGAATTCATGGAAGACCTGGGGGAAAA-3’
MIS12-1,120-ECOR1-F	5’-GAGAGAATTCATGTCTGTGGATCCAATGACC-3’
MIS12-31,205-SAL1-R	5’-GAGAGTCGACTTAAGATATTTTCAGTCGTTTCGA-3’
